# Gibberellin Homeostasis Perturbation Alters Sugar Metabolism and Developmental Traits in *Agapanthus praecox* subsp. *orientalis*

**DOI:** 10.3390/plants15172699

**Published:** 2026-09-02

**Authors:** Jianhua Yue, Tingting Fang, Yan Dong, Changmei Du, Xinran Cai, Feiyang Zhang, Yi Wang, Wenjing Luo, Yifan Liu, Yunshu Yang, Jiye Li, Hongwei Fan, Shoufu Gong

**Affiliations:** 1 School of Horticulture, Xinyang Agriculture and Forestry University, Xinyang 461000, China; 2 School of Forestry, Xinyang Agriculture and Forestry University, Xinyang 461000, China

**Keywords:** *Agapanthus praecox* subsp. *orientalis*, gibberellins, source–sink dynamics, sugar metabolism, developmental plasticity

## Abstract

Gibberellins (GAs) are essential hormones that regulate plant growth and development, yet the downstream metabolic pathways through which they modulate phenotypic plasticity remain poorly understood. Here, we investigated the GA-mediated regulatory network in *Agapanthus praecox* subsp. *orientalis* by integrating phenotypic, transcriptomic, and targeted metabolomic analyses across three complementary perturbation strategies: gradient paclobutrazol (PAC) treatments, *GA20ox* RNA interference (RNAi) lines, and exogenous GA_4_ rescue. PAC suppressed vegetative growth dose-dependently and completely arrested floral development at 200 mg·L^−1^, while increasing soluble sugar and starch contents; plants treated with it in the first year displayed enhanced vegetative growth in the following season, suggesting a possible carbon legacy effect. *GA20ox* silencing caused dwarfism and flowering failure, which were largely rescued by GA_4_. Exogenous GA_4_ restored bioactive GA pools to above wild-type levels without recovering endogenous synthesis, indicating functional compensation. Transcriptomic and qRT-PCR analyses revealed that sugar metabolism-related genes and metabolites underwent the most pronounced changes among all tested categories; these changes were partially reversed by GA_4_, though certain catabolic genes remained suppressed, revealing hierarchical regulatory logic. Collectively, these findings indicate that GA is closely associated with growth and carbohydrate allocation, and that the GA–sugar metabolic axis represents a major downstream component in developmental plasticity. This study provides species-specific insights into GA-mediated growth regulation and offers implications for ornamental crop management.

## 1. Introduction

Gibberellins (GAs) are a class of diterpenoid hormones that play essential roles in plant growth and development, including seed germination, stem elongation, leaf expansion, floral transition, and fruit development [1,2,3,4]. Among the 136 known GA molecules, only a few (GA_1_, GA_3_, GA_4_, and GA_7_) are biologically active [5,6]; these bioactive GAs, together with their deactivated catabolites GA_8_ and GA_34_, are generated during GA turnover [6]. They bind to the receptor GIBBERELLIN-INSENSITIVE DWARF1 (GID1), triggering the degradation of DELLA proteins, which are the core repressors of GA signaling [7,8,9]. These proteins interact with multiple transcription factors, such as phytochrome-interacting factors (PIFs) and BRASSINAZOLE RESISTANT 1 (BZR1), to regulate cell proliferation and expansion, conferring a high degree of developmental plasticity [10].

Given the pivotal role of GAs in regulating plant height, genetic manipulation of GA metabolism has profoundly reshaped global agriculture [11]. In the mid-20th century, semi-dwarf mutants (*ga20ox*) in wheat and rice significantly reduced plant height, increased lodging resistance, and improved harvest index, leading to the “Green Revolution” [12,13,14]. Concurrently, chemical inhibitors targeting GA biosynthesis have proven effective in modulating plant architecture: paclobutrazol (PAC) and uniconazole, which block key oxidases in the early GA biosynthetic steps, suppress stem elongation and are widely used in fruit tree dwarfing, ornamental plant shape control, and turf management [15]. However, the dose–response of such inhibitors has a critical threshold: excessive suppression not only causes severe growth arrest but may also disturb reproductive development and trigger a fundamental rerouting of carbon allocation [16]. Understanding the dose-dependent effects of GA perturbation is therefore of both theoretical and practical importance.

Beyond growth regulation, GAs play complex roles in plant responses to abiotic stress [17]; under drought, salinity, or cold stress, plants often reduce GA content or signaling to suppress growth and reallocate resources to defense [18]. Exogenous GA_3_ application can partially alleviate stress-induced damage by modulating osmotic adjustment, antioxidant enzyme activities, and photosynthetic efficiency [19]. Moreover, GA crosstalks extensively with abscisic acid (ABA), ethylene (ET), and brassinosteroids (BRs) to coordinate the growth–defense trade-off [17,20]. Thus, GA functions not only as an accelerator of growth but also as a key node for adjusting developmental strategies under stress [20].

Increasing evidence indicates that GA-mediated growth regulation is closely linked to primary metabolism [21]. Sucrose metabolism regulates plant growth, stress adaptation, and yield by providing carbon resources and sugar signals that integrate hormonal and defense pathways [22,23]. Recent studies have uncovered intercellular sugar signaling crosstalk, signaling functions of sucrose metabolic enzymes, and metabolic engineering approaches for higher yield and disease resistance [24]. As a central regulator of source–sink dynamics, GA plays a key role in regulating the allocation of photoassimilates from source organs to sink tissues by enhancing sink strength and demand [25]. GA and sugar signaling engage in bidirectional crosstalk: sugars serve both as metabolites and signals that influence GA biosynthesis and signaling, while GA reciprocally modulates key enzymes in carbohydrate metabolism (e.g., α-amylase) to control carbohydrate mobilization and utilization [26,27]. This hormone–sugar interplay operates during seed germination, seedling establishment, tuberization, and fruit development, enabling plants to flexibly adjust growth rates under varying carbon availability [24]. Similar GA–sugar crosstalk has been observed in the sprouting regeneration of *Pinus yunnanensis*, where exogenous GA_3_ promoted higher sprout numbers accompanied by elevated trehalose and glucose levels [28]. However, the specific pathways through which GA modulates sugar metabolism in association with growth and phenotypic plasticity remain to be fully elucidated in ornamental species.

*Agapanthus praecox* subsp. *orientalis* is a perennial herb native to South Africa, valued globally as an ornamental plant for its elegant inflorescence and long flowering period [29,30,31]. Previous studies have demonstrated that GA plays an important role in pedicel elongation and flowering time regulation in *A. praecox* subsp. *orientalis*: exogenous GA markedly promotes pedicel elongation and induces early flowering, with GA_4_ being the major bioactive form responsible for floral development [32]. Transcriptomic and proteomic analyses have revealed that carbohydrate metabolism provides the nutritional foundation for floral transition, while GA and auxin signaling jointly regulate cell elongation and proliferation in the pedicel [33,34,35]. *GA20ox* was confirmed as the key enzyme controlling GA_4_ biosynthesis [36]. Nevertheless, systematic studies on how GA modulates primary metabolism to affect growth and development in this species are still lacking. We previously found that both exogenous PAC treatment and RNAi-mediated suppression of *GA20ox* significantly altered growth and development in *A. praecox* subsp. *orientalis* [36]. However, the specific molecular network through which GA regulates development in this species has not been systematically explored.

Hence, this study employed PAC gradient treatments, *GA20ox*-RNAi transgenic lines, and exogenous GA rescue experiments, integrating phenotypic, hormonal metabolomic, transcriptomic, and targeted sugar metabolomic approaches to dissect the GA-mediated regulatory network. Our specific objectives were as follows: (1) to characterize the dose-dependent effects of PAC on vegetative growth, reproductive development, and carbon partitioning, including source–sink reallocation and cross-seasonal carry-over effects; (2) to assess growth inhibition caused by *GA20ox* silencing and its rescue by exogenous GA; (3) to identify key pathways responsive to GA perturbation via KEGG enrichment; (4) to evaluate whether sugar metabolism functions as a major downstream component associated with GA-mediated regulation of growth and phenotypic plasticity. Overall, our findings provide species-specific insights into the role of GA in regulating growth and metabolism in *A. praecox* subsp. *orientalis*, with potential implications for ornamental crop management.

## 2. Results

### 2.1. Dose-Dependent Effects of PAC on Vegetative Growth, Flowering, and Carbohydrate Metabolism

**Figure 1:**
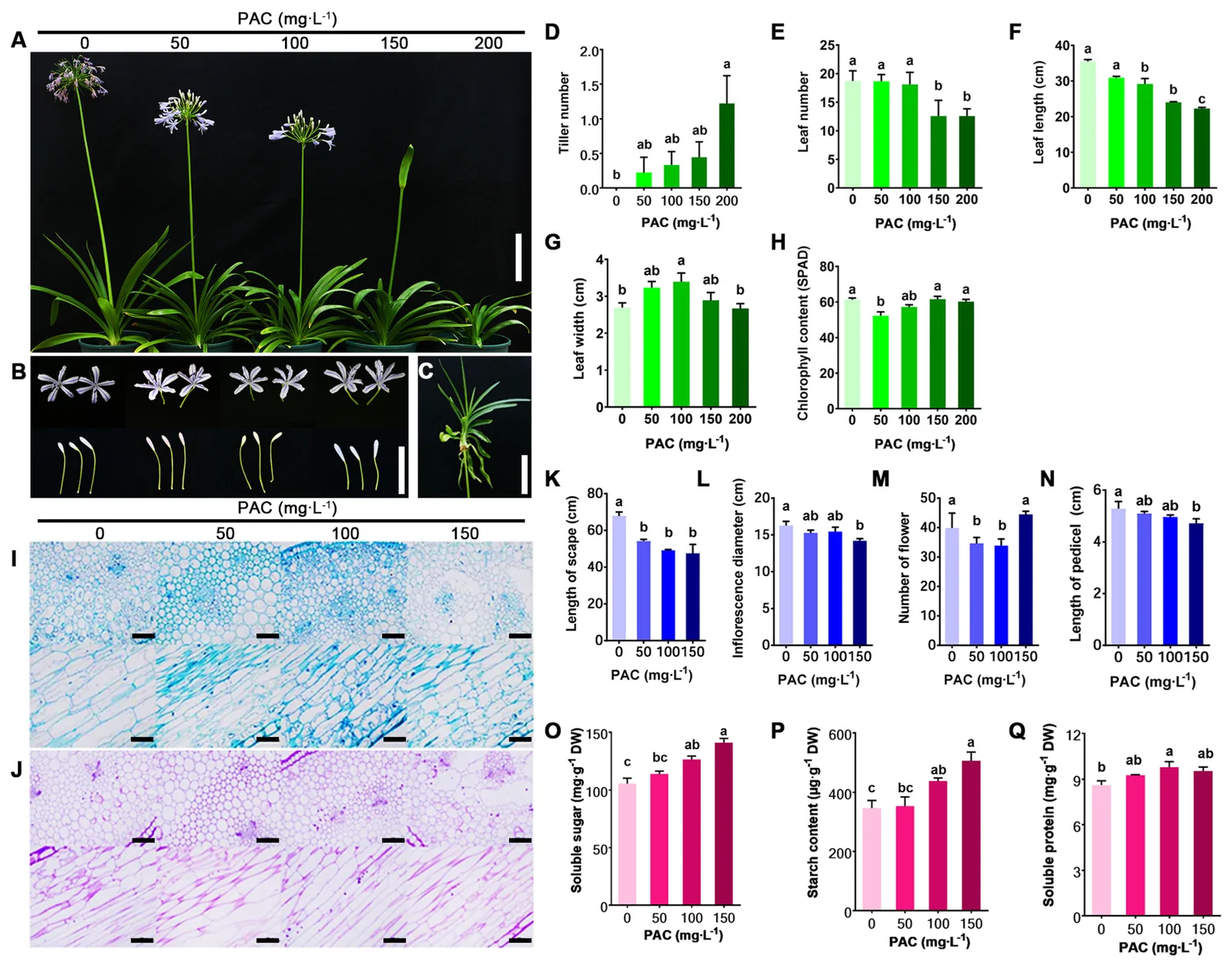
Dose-dependent effects of PAC on plant growth and carbohydrate metabolism. (**A**) Dose-dependent (0, 50, 100, 150, and 200 mg·L^−1^) effects of PAC on plant morphology; scale bar = 20.0 cm. (**B**) Dose-dependent (0, 50, 100, and 150 mg·L^−1^) effects of PAC on flowering; scale bar = 5.0 cm. (**C**) Effects of 200 mg·L^−1^ PAC on flowering; scale bar = 10.0 cm. (**D**) Tiller number per plant; data are presented as mean ± SE; *n* = 3, different lowercase letters above the bars indicate significant differences at *p* < 0.05, with the same applying hereinafter. (**E**) Leaf number per plant. (**F**) Leaf length. (**G**) Leaf width. (**H**) Relative chlorophyll content (SPAD units). (**I**) Morphological differences in samples from pedicel by hematoxylin staining. The micromorphology of transverse and longitudinal sections was provided. Scale bars = 100 μm. (**J**) PAS staining of pedicel. Scale bars = 100 μm. (**K**) Scape length. (**L**) Inflorescence diameter. (**M**) Number of flowers per inflorescence. (**N**) Pedicel length. (**O**) Soluble sugar content in pedicel. (**P**) Starch content in pedicel. (**Q**) Soluble protein content in pedicel.

As PAC concentration increased, plant height (leaf length and scape height) progressively decreased and flowering time was delayed (Figure 1A), accompanied by a gradual reduction in floral organ size (Figure 1B). Notably, at 200 mg·L^−1^ PAC, reproductive development was completely arrested, with floral primordia converting into vegetative leaf-like buds (Figure 1C), indicating that excess PAC inhibits floral transition and promotes vegetative proliferation. Tiller number increased with rising PAC concentration (Figure 1D), while leaf number and length declined progressively, confirming a pronounced dwarfing effect (Figure 1E,F). Leaf width did not change monotonically along the PAC gradient; the maximum value occurred at 100 mg·L^−1^ PAC, significantly higher than that for the CK (0 mg·L^−1^) and 200 mg·L^−1^ groups (Figure 1G). SPAD values dipped at low PAC concentration then stabilized at the control level for medium-high doses (Figure 1H). Notably, although cell sizes were relatively uniform in cross-section, longitudinal axis lengths varied among pedicels (Figure 1I). Moreover, starch granule accumulation in pedicels increased progressively with rising PAC dosage (Figure 1J), scape length exhibited a significant dose-dependent reduction (Figure 1K), and inflorescence diameter and pedicel length gradually decreased as PAC concentration increased (Figure 1L–N). Soluble sugar (Figure 1O) and starch (Figure 1P) contents increased consistently across the entire gradient, while soluble protein content peaked at 100 mg·L^−1^ PAC and dipped slightly at 150 mg·L^−1^, lacking a simple dose-dependent upward trend (Figure 1Q). Collectively, these results demonstrate that PAC exerts dose-dependent effects on plant development and carbohydrate metabolism.

### 2.2. Carry-Over Effects of PAC on Growth in the Subsequent Season

**Figure 2:**
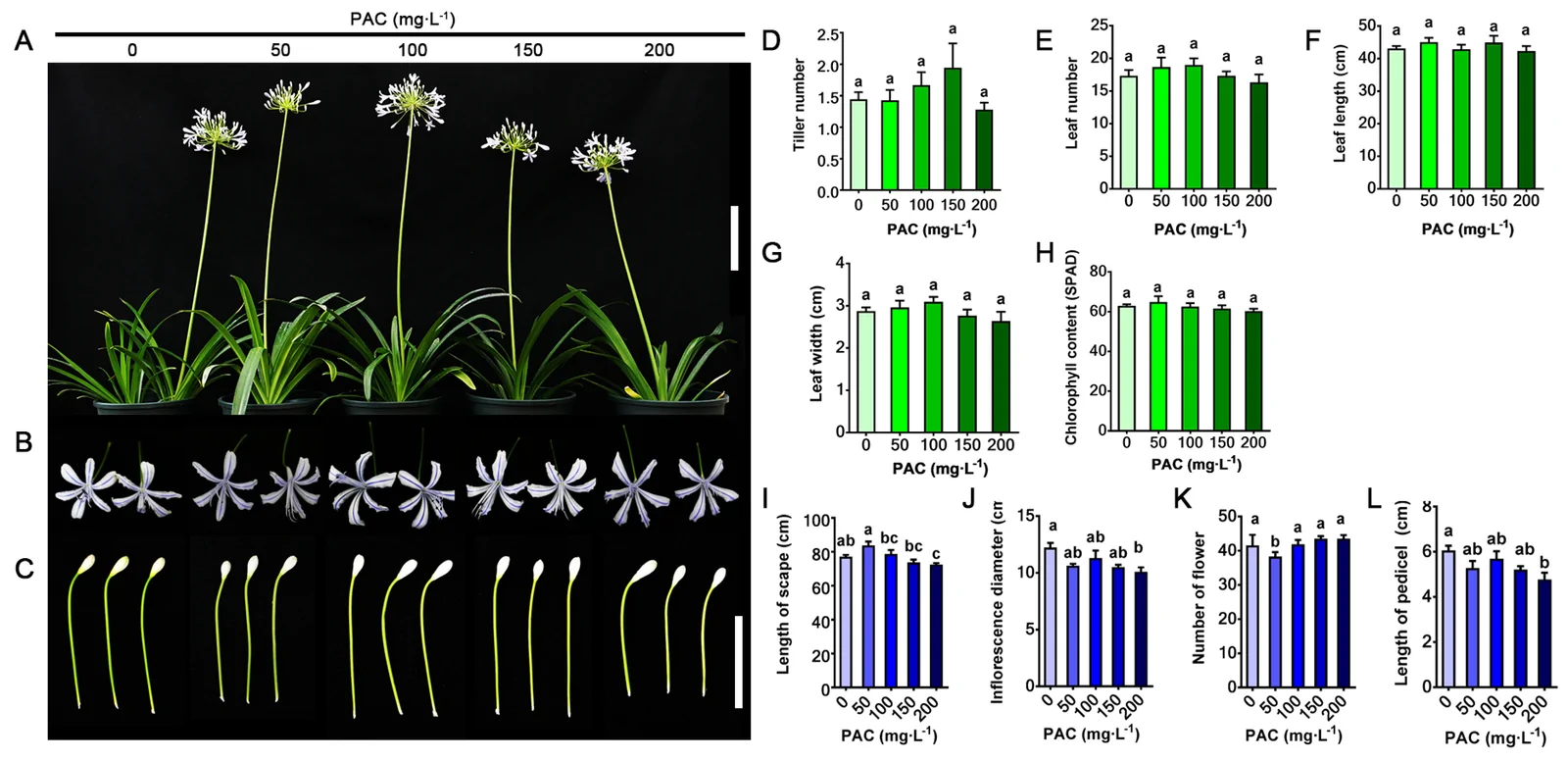
Carry-over effects of PAC treatment on agronomic traits in the second year. Plants were treated with increasing PAC concentrations (0, 50, 100, 150, 200 mg·L^−1^) in the previous year, and all measurements were taken in the following growing season after PAC withdrawal. (**A**) Dose-dependent effects of PAC on plant phenotype in the next season; scale bar = 20.0 cm. (**B**,**C**) Morphology of florets and pedicels; scale bar = 5.0 cm. (**D**) Tiller number; data are presented as mean ± SE; *n* = 3, different lowercase letters above the bars indicate significant differences at *p* < 0.05, with the same applying hereinafter. (**E**) Leaf number. (**F**) Leaf length. (**G**) Leaf width. (**H**) Relative chlorophyll content. (**I**) Scape length. (**J**) Inflorescence diameter. (**K**) Number of flowers per inflorescence. (**L**) Pedicel length.

In the second year following PAC withdrawal, the previously observed growth inhibition not only was alleviated but also showed altered effects on most vegetative parameters (Figure 2). Plants that had received PAC concentrations (50 and 100 mg·L^−1^) in the prior season exhibited enhanced overall plant stature (Figure 2A), and the florets and pedicels appeared more robust (Figure 2B,C). Moreover, tiller number increased with rising PAC concentration and reached a maximum at 150 mg·L^−1^ (Figure 2D), and leaf number also peaked at 150 mg·L^−1^ (Figure 2E). Leaf length showed little change (Figure 2F), whereas leaf width exhibited an increasing trend with PAC dosage and peaked at 100 mg·L^−1^ (Figure 2G). Relative chlorophyll content also showed a slight increase followed by a slight decrease (Figure 2H). Most of these morphological parameters did not show statistically significant differences (*p* > 0.05). Scape length in the 50 mg·L^−1^ group increased significantly with higher PAC concentrations (Figure 2I). However, reproductive traits, including inflorescence diameter (Figure 2J), flower number per inflorescence (Figure 2K), and pedicel length (Figure 2L), did not show clear dose-dependent changes. Collectively, these results indicate that PAC treatment in the previous year exerted a different effect on vegetative growth in the following season.

### 2.3. Exogenous GA Rescues the Growth Defects and Reprograms the Hormonal Metabolism in RNAi Lines

**Figure 3:**
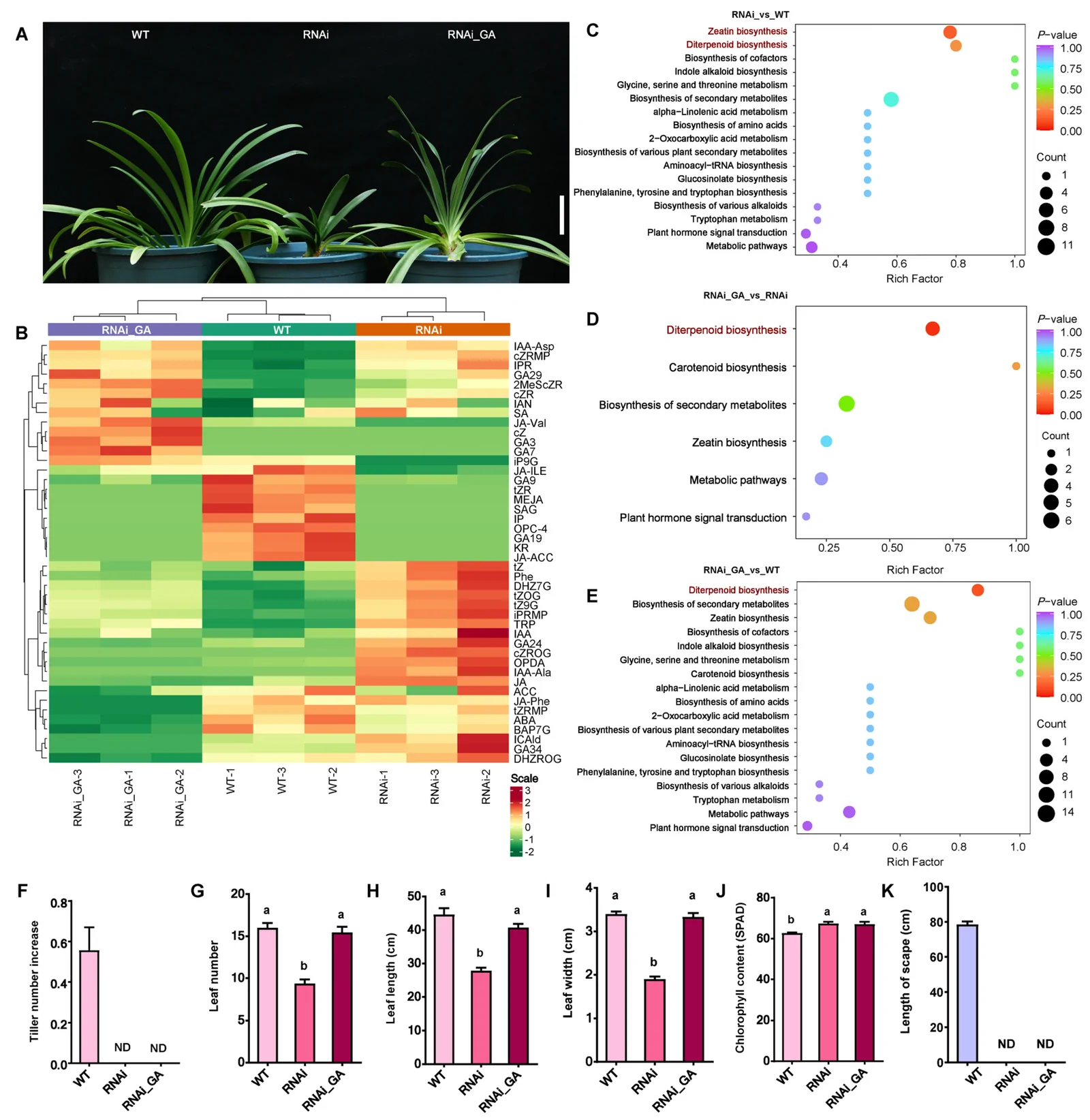
Phenotypic changes, targeted hormonal metabolomics, and KEGG pathway enrichment analyses among WT, RNAi, and RNAi_GA. (**A**) Representative phenotypes of WT, gene-silenced (RNAi), and exogenous gibberellin-supplemented silenced lines (RNAi_GA). Bar = 10 cm. (**B**) Heatmap of DAMs based on targeted metabolomics profiling across the three groups. (**C**) KEGG pathway enrichment analysis of DAMs between WT and RNAi, (**D**) RNAi and RNAi_GA, and (**E**) RNAi_GA and WT. The color scale represents the significance level. (**F**) Tiller number increase; data are presented as mean ± SE; *n* = 3, different lowercase letters above the bars indicate significant differences at *p* < 0.05, with the same applying hereinafter. (**G**) Leaf number. (**H**) Leaf length. (**I**) Leaf width. (**J**) Relative chlorophyll content. (**K**) Scape length. ND indicates not detected.

RNAi plants exhibited pronounced growth retardation, including reduced plant height and smaller leaf size, compared with WT. Notably, RNAi_GA substantially restored normal growth (Figure 3A), indicating that the silencing-induced developmental defects were mainly attributable to impaired GA homeostasis or signaling.

Targeted hormonal metabolomics revealed a distinct hormonal accumulation pattern in RNAi relative to WT, with significant alterations in multiple phytohormone classes (Figure 3B and Supplementary Table S1). Notably, the hormonal profile of RNAi_GA shifted markedly toward that of WT, suggesting that exogenous GA effectively rebalanced the endogenous hormonal network, which was highly consistent with the phenotypic recovery.

KEGG pathway enrichment analysis was used to identify the key affected metabolic pathways. In the WT vs. RNAi comparison (Figure 3C), the differentially accumulated metabolites (DAMs) were significantly enriched in pathways related to diterpenoid biosynthesis (the core GA biosynthetic pathway) and zeatin biosynthesis, confirming that gene silencing severely disrupted GA metabolism and its crosstalk with other hormones. In the RNAi vs. RNAi_GA comparison (Figure 3D), the same set of hormone-related pathways, especially diterpenoid biosynthesis, was significantly enriched, indicating that exogenous GA supplementation actively reversed the silencing-induced metabolic disturbances. More importantly, in the RNAi_GA vs. WT comparison (Figure 3E), most of the previously enriched pathways, including those involved in GA metabolism, were enriched once again, further validating that exogenous GA treatment restored the metabolic state to a level comparable to that of the WT, primarily through changes in diterpenoid biosynthesis.

The increased tiller number was observed only in WT, and was not recorded for RNAi and RNAi_GA (Figure 3F). Leaf number decreased significantly in RNAi plants and was restored to the WT level in RNAi_GA (Figure 3G); leaf length, leaf width, and the length of leaf cells showed the same pattern: they were significantly reduced in RNAi and markedly recovered in RNAi_GA (Figure 3H,I and Supplementary Figure S1). Chlorophyll content was significantly higher in RNAi and RNAi_GA than in WT, while RNAi and RNAi_GA did not differ statistically (Figure 3J). Scape length was measurable only in WT, while RNAi lines exhibited failed flowering (Figure 3K and Supplementary Figure S2).

In summary, these results confirm that the target gene is essential for normal growth by regulating GA metabolism: silencing it disrupts hormone homeostasis and inhibits growth, whereas exogenous GA application largely rescues most phenotypic defects, including leaf size, by restoring key metabolic pathways. However, tillering and flowering were not recovered, suggesting that certain traits may involve additional regulatory mechanisms. Overall, GA serves as a key effector that can be externally manipulated to modulate plant architecture.

### 2.4. Targeted Metabolomics Reveals GA Flux Reprogramming Among WT, RNAi, and RNAi_GA

**Figure 4:**
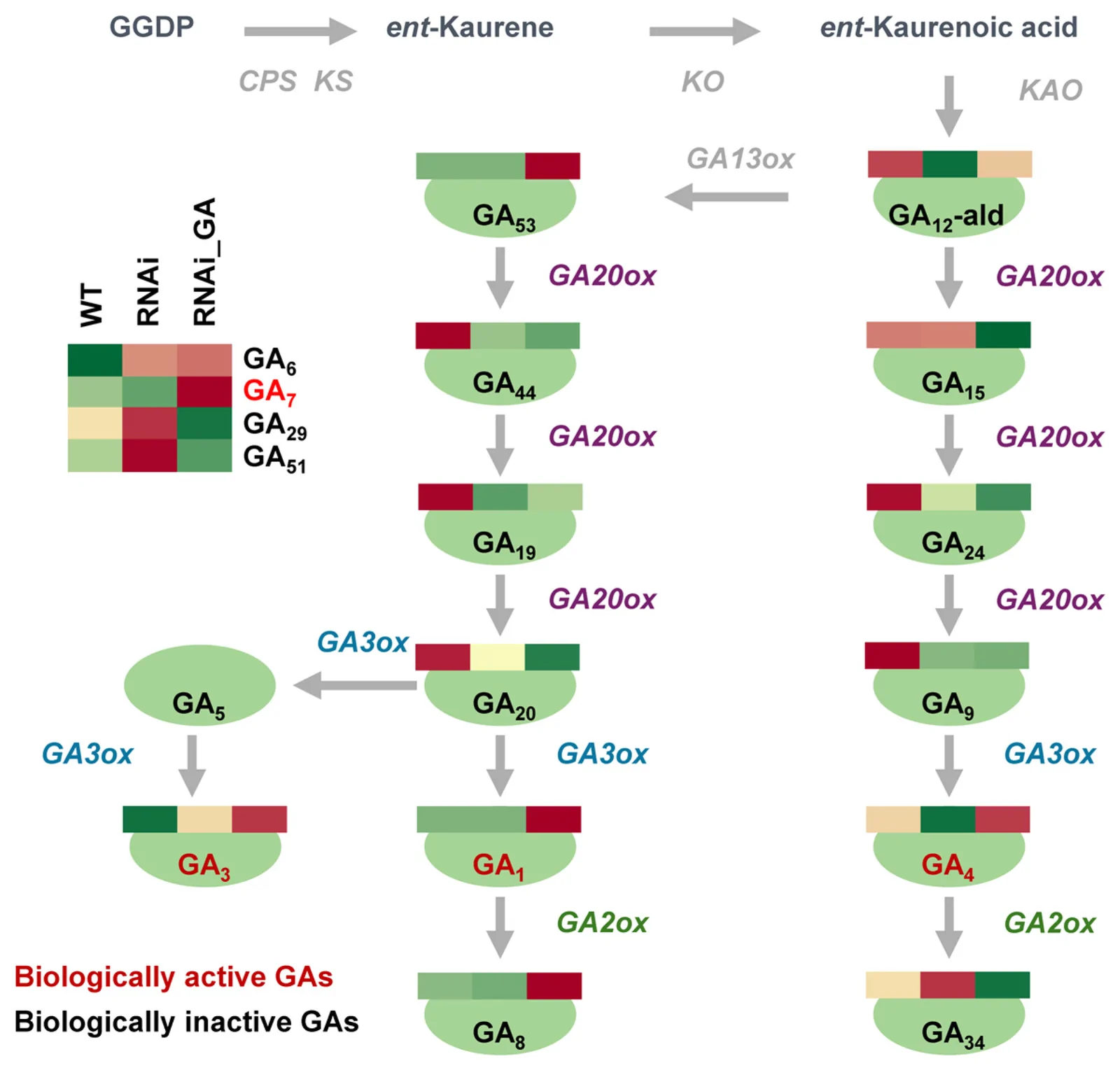
Targeted metabolomic heatmap of the GA biosynthetic pathway in WT, RNAi, and RNAi_GA. The color scale from green to red indicates low to high relative metabolite contents.

A total of 17 GAs were detected across all groups (Figure 4, Supplementary Table S2). Compared with WT, in the RNAi lines, the intermediates of GAs, including GA_12_-aldehyde (GA_12_-ald), GA_24_, GA_9_, GA_44_, GA_19_, and GA_20_, were significantly reduced (*p* < 0.05), and the bioactives GA_4_ and GA_7_ also decreased significantly (*p* < 0.05). Meanwhile, the catabolites GA_34_, GA_51_, and GA_29_, and the bioactive GA_3_, were all significantly elevated relative to the WT (*p* < 0.05). These changes collectively explained the growth inhibition observed in the RNAi plants.

Exogenous GA application (RNAi_GA) exhibited a complex pattern of change. The endogenous pathway remained irreversibly blocked, and the set of downstream intermediates (GA_15_, GA_24_, GA_9_, GA_44_, GA_19_, and GA_20_) did not recover; their contents remained significantly below WT values (*p* < 0.05) and showed no improvement over the RNAi group. These metabolites were therefore not rescued. However, the bioactive GAs, including GA_1_, GA_3_, GA_4_, and GA_7_, were effectively replenished: their concentrations in RNAi_GA were significantly higher than WT and RNAi levels (*p* < 0.05). The exogenous supply also triggered enhanced turnover metabolism, as reflected by further increases in GA_8_ and GA_53_ compared with the RNAi group.

Collectively, these results indicate that silencing of *GA20ox* blocked the endogenous flux from early precursors toward downstream intermediates and the bioactive GA_4_. Exogenous GA application bypassed this block specifically for GA_4_ and GA_7_, but it failed to restore the endogenous intermediate chain, while simultaneously accelerating catabolic and shunt metabolism.

### 2.5. GO and KEGG Enrichment Analyses Reveal Restoration of Metabolism

We performed RNA-seq analysis on WT, RNAi, and RNAi_GA lines, generating a total of 56.82–68.04 million clean reads per sample, with Q30 > 98.5% (Supplementary Table S3). De novo assembly using Trinity yielded 228,325 transcripts with an N50 of 1709 bp (Supplementary Table S4), confirming the high quality of the transcriptome assembly. Principal component analysis (PCA) revealed clear separation among the three groups along PC1 (Supplementary Figure S3), indicating that GA perturbation was the major source of transcriptional variation, with pairwise sample correlations within each treatment group consistently high (>0.95), confirming biological replicate reproducibility (Supplementary Figure S4). Differential expression analysis identified 23,275, 9305, and 20,839 differentially expressed genes (DEGs) in the RNAi vs. WT, RNAi_GA vs. RNAi, and RNAi_GA vs. WT comparisons, respectively (Supplementary Figure S5).

These transcriptomic data provide a global view of the molecular responses to GA perturbation and rescue, and serve as the basis for subsequent functional enrichment and pathway analyses. The changes in 20 mRNA transcripts assessed by qRT-PCR were similar (*R* = 0.862; *p* < 0.05) to those based on RNA-Seq analysis, suggesting the validity of the RNA-Seq data (Supplementary Figure S6)

**Figure 5:**
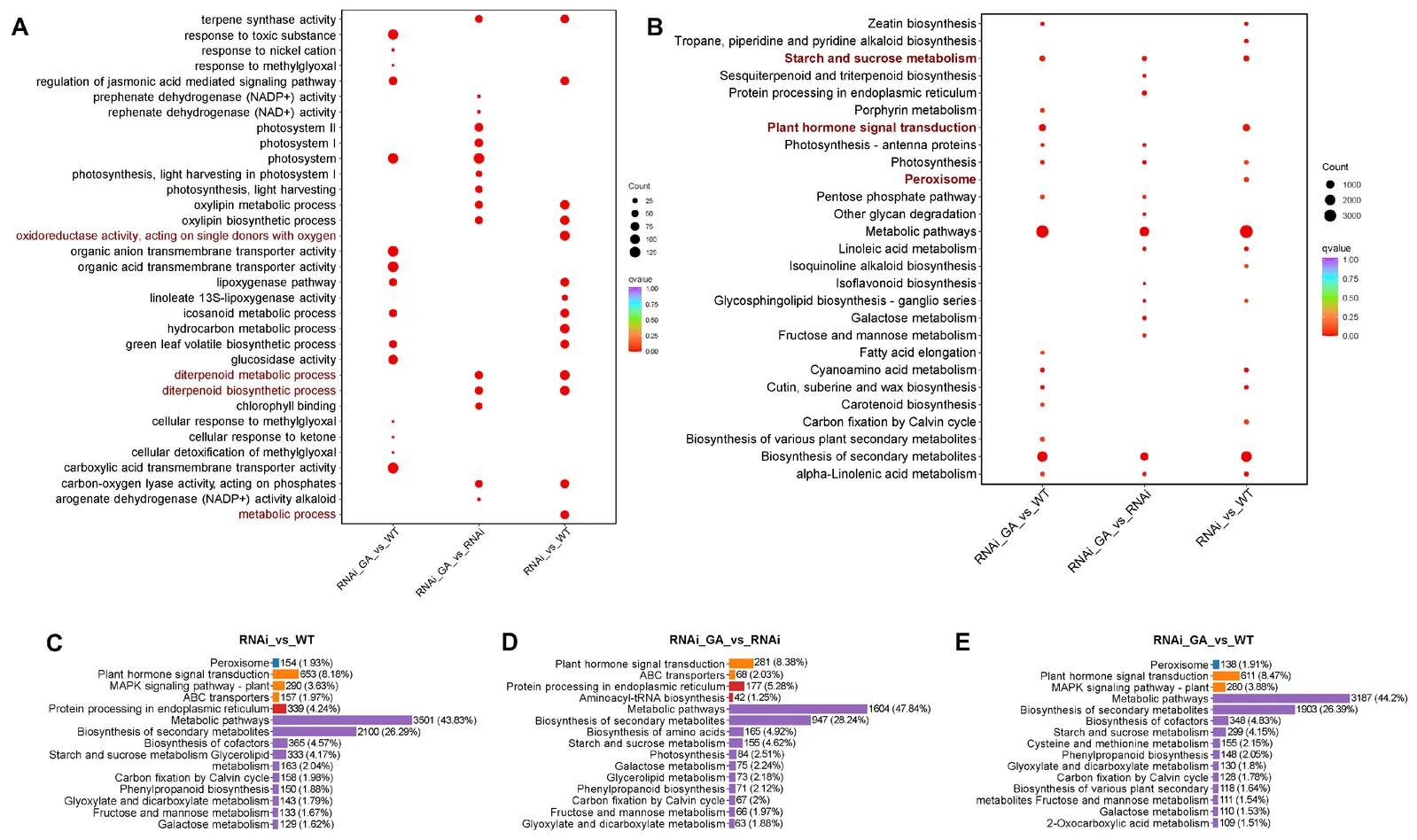
GO and KEGG pathway enrichment analyses among WT, RNAi, and RNAi_GA. (**A**) GO enrichment analyses. (**B**) KEGG pathway enrichment analyses. (**C**) KEGG pairwise comparisons of RNAi vs. WT, (**D**) RNAi_GA vs. RNAi, and (**E**) RNAi_GA vs. WT.

GO and KEGG pathway enrichment analyses were performed on DEGs among the three groups (Figure 5). In the RNAi vs. WT comparison, the GO terms oxidoreductase activity and metabolic process were significantly enriched (Figure 5A). The terms diterpenoid metabolic process and diterpenoid biosynthetic process were significantly enriched in both the RNAi vs. WT and RNAi_GA vs. RNAi comparisons, whereas no significant enrichment of these terms was detected in the RNAi_GA vs. WT comparison. KEGG pathway enrichment analysis (Figure 5B) showed that the peroxisome pathway was significantly enriched in the RNAi vs. WT comparison, the plant hormone signal transduction pathway was significantly enriched in both the RNAi vs. WT and RNAi_GA vs. WT comparisons, and the starch and sucrose metabolism pathway was significantly enriched in all three pairwise comparisons.

In the RNAi vs. WT comparison (Figure 5C), the most significantly enriched KEGG terms included those related to oxidative stress, plant hormone signal transduction, and carbohydrate metabolism, indicating that gene silencing triggered a broad stress response, hormonal imbalance, and substantial alterations in metabolic flux.

In the RNAi_GA vs. RNAi comparison (Figure 5D), the plant hormone signal transduction pathway was significantly enriched, whereas the peroxisome pathway showed no significant difference, suggesting that exogenous GA rebalanced the hormonal network but did not effectively rescue the stress response.

In the RNAi_GA vs. WT comparison (Figure 5E), the oxidative stress-related KEGG terms and hormone signaling categories exhibited a pronounced shift toward WT-like levels, confirming that the metabolic and transcriptional status of the RNAi_GA lines had largely returned to that of the WT.

The KEGG pathway enrichment results (Figure 5B) supported these observations, showing consistent trends in the corresponding metabolic and signaling pathways across the three comparisons, implying that exogenous GA rebalanced both the hormonal network and carbohydrate metabolism.

Collectively, these results indicate that diterpenoid metabolism could be effectively modulated by exogenous GA application. The phenotypic differences and stress tolerance alterations among the three groups were jointly governed by coordinated changes in GA biosynthesis, oxidative stress responses, carbohydrate metabolism, and hormone signal transduction pathways.

### 2.6. qRT-PCR Reveals Sugar Metabolism as the Primary Pathway Mediating GA-Dependent Phenotypes

**Figure 6:**
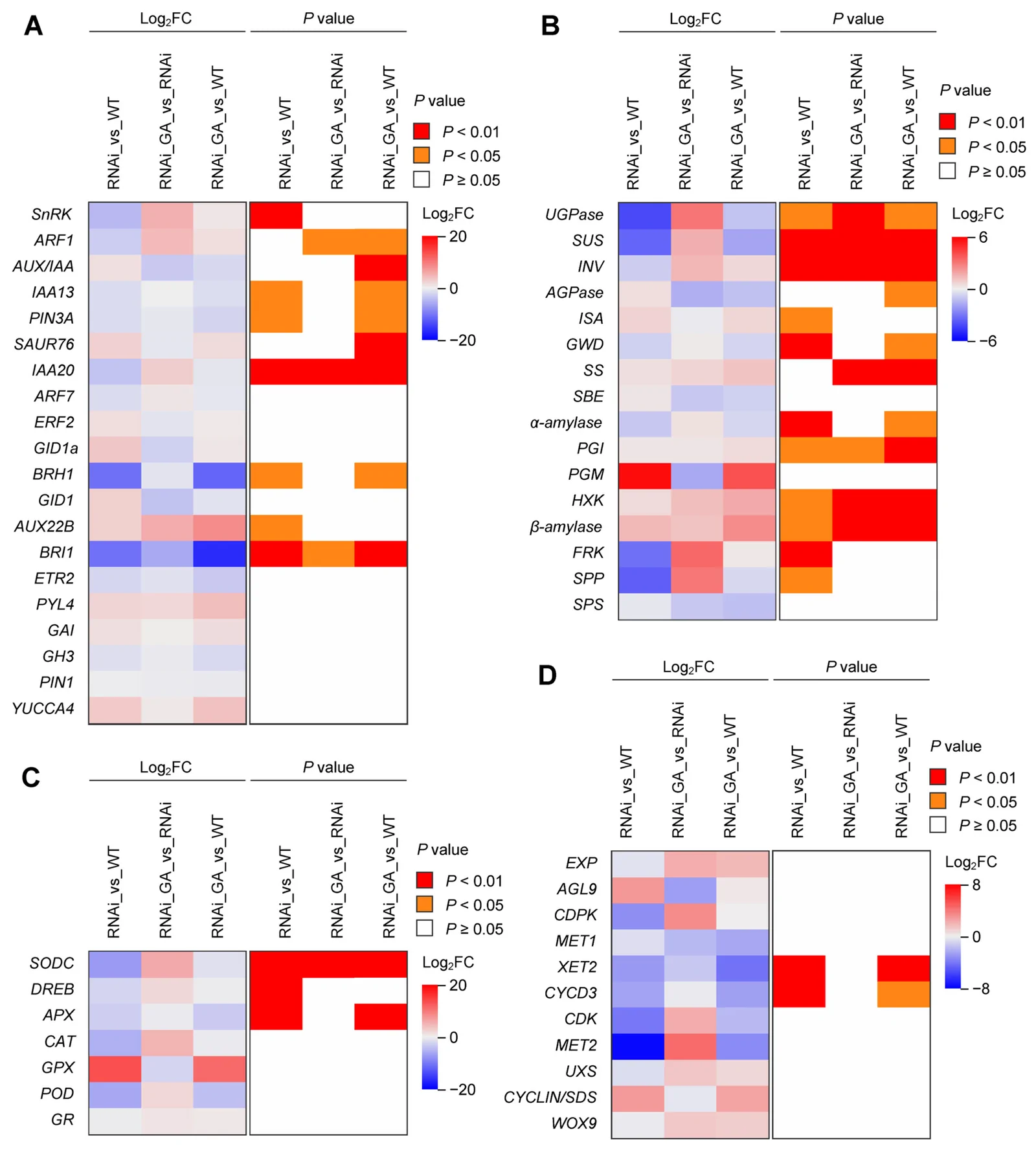
qRT-PCR validation of DEGs in WT, RNAi, and RNAi_GA lines. (**A**) Expression patterns of DEGs involved in phytohormone biosynthesis and metabolism, (**B**) carbohydrate metabolism, (**C**) stress response, and (**D**) development.

qRT-PCR was performed on selected genes across WT, RNAi, and RNAi_GA lines to validate the transcriptomic trends and identify key downstream effectors (Figure 6, Supplementary Figure S7).

For phytohormone-related genes (Figure 6A), most hormone signaling components, including auxin-related genes (*ARF1*, *IAA20*, *ARF7*), were downregulated in RNAi and upregulated in RNAi_GA; notably, the BR marker genes (*BRH1*, *BRI1*) were significantly downregulated in both RNAi and RNAi_GA lines. Remarkably, carbohydrate metabolism-related genes (Figure 6B), including those associated with sugar metabolism (*UGPase*, *SUS*, *INV*, *PGI*, *HXK*, and *β-amylase*), exhibited the most significant changes among all comparisons, while *GWD* and *α-amylase* also showed significant alterations compared with WT. Notably, the expression of *AGPase*, *ISA*, *GWD*, and *α-amylase* failed to recover with exogenous GA rescue. For stress-responsive genes, oxidative stress-responsive genes (*SODC*, *DREB*, *APX*, *CAT*, *POD*, and *GR*) were downregulated in RNAi lines (Figure 6C), and *XET2* and *CYCD3* were significantly downregulated in RNAi and RNAi_GA (Figure 6D). These results indicate that gene silencing severely compromised basal energy and carbon flux, and that carbohydrate metabolism was sensitive to GA biosynthesis deficiency.

In the RNAi_GA vs. RNAi comparison, exogenous GA application effectively reversed the expression of a subset of sugar metabolism-related genes, whereas the hormone-, stress- and developmental-related genes remained largely unrecovered.

Collectively, the qRT-PCR results show that sugar metabolism exhibited the most pronounced changes among the tested pathways, and suggest that GA is associated with the coordination of hormone signaling and carbohydrate metabolism, which may contribute to the regulation of growth and developmental plasticity.

### 2.7. Sugar Metabolism Is Closely Associated with the Response to GA Perturbation

**Figure 7:**
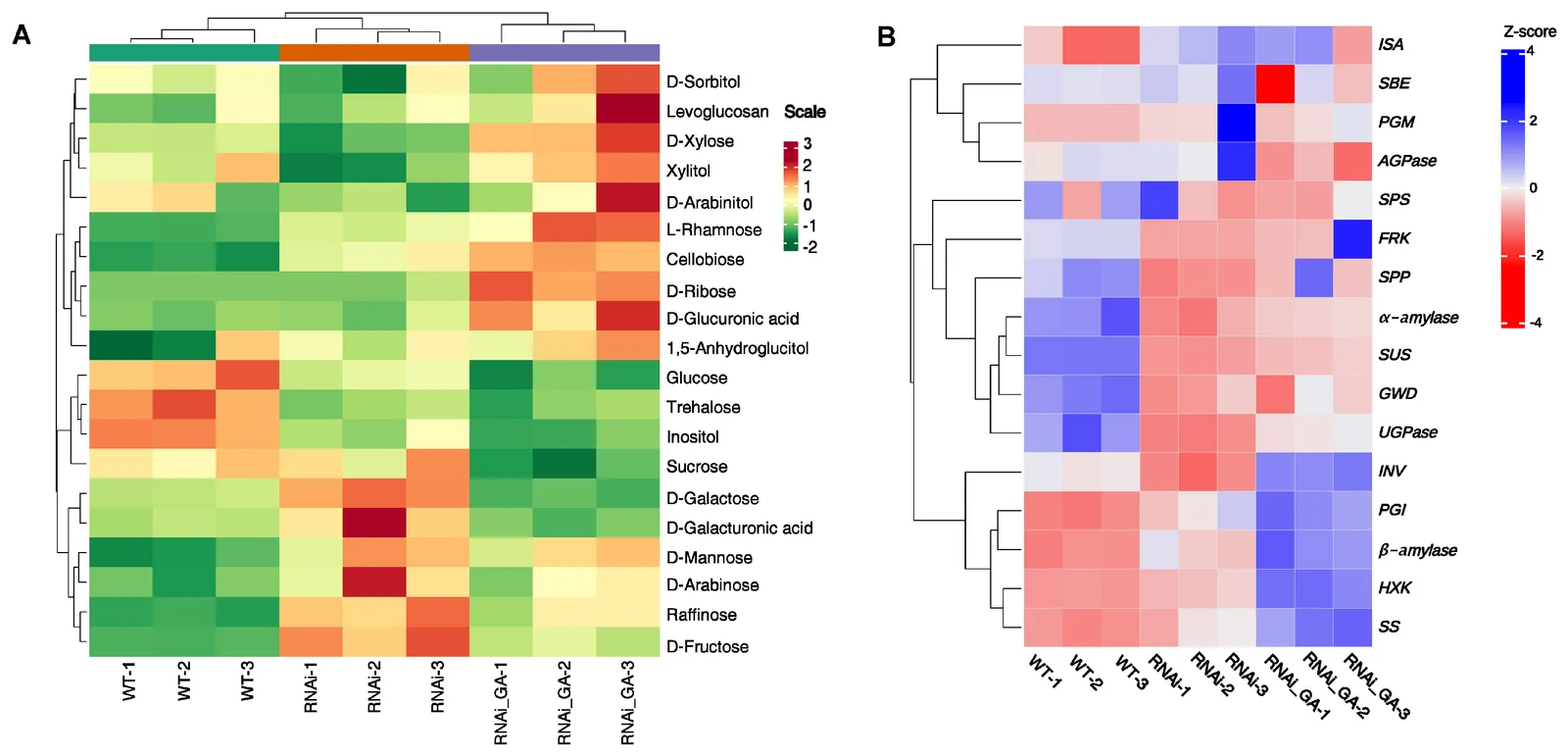
Heatmap analysis of sugar metabolites and related metabolic enzymes across WT, RNAi, and RNAi_GA. (**A**) Relative abundance of 20 sugar metabolites. (**B**) Expression levels of 16 key enzymes involved in sugar metabolism, including starch synthesis (*AGPase*, *SS*, *SBE*, *ISA*) and starch degradation (*α-amylase*, *β-amylase*, *GWD*), sucrose metabolism (*SPS*, *SPP*, *INV*, *SUS*), glycolysis, and the pentose phosphate pathway (*PGI*, *HXK*, *FRK*, *PGM*), and other related enzymes (*UGPase*). The color scale indicates relative abundance or expression levels after normalization across samples; red represents higher levels and green/blue represents lower levels.

The relative abundance of 20 sugar metabolites (Figure 7A, Supplementary Table S5) and expression levels of 16 key enzyme genes involved in starch synthesis and degradation, sucrose metabolism, glycolysis, and the pentose phosphate pathway (Figure 7B) were compared across WT, RNAi, and RNAi_GA lines.

In the RNAi vs. WT comparison, different sugar metabolites exhibited distinct alteration patterns: glucose, trehalose, and inositol were significantly decreased, while D-galactose, D-galacturonic acid, D-mannose, D-arabinose, raffinose, and D-fructose were significantly increased. Upon exogenous GA application (RNAi_GA vs. RNAi), most metabolites showed a recovery trend. D-sorbitol, levoglucosan, D-xylose, xylitol, D-arabinitol, L-rhamnose, cellobiose, D-ribose, and D-glucuronic acid were significantly elevated compared with RNAi; in contrast, sucrose was significantly decreased, and D-galactose, D-galacturonic acid, D-mannose, D-arabinose, raffinose, and D-fructose showed reduced levels relative to those for RNAi (Figure 7B).

In the RNAi vs. WT comparison, the expression changes in DEGs also exhibited distinct patterns. *ISA*, *SBE*, *PGM*, and *AGPase* were downregulated, although *SBE* showed marked recovery upon exogenous GA application (RNAi_GA vs. RNAi); in contrast, *SPS*, *FRK*, *SPP*, *α-amylase*, *SUS*, *GWD*, and *UGPase* were upregulated in RNAi compared with WT, and their expression levels remained relatively high even after exogenous GA treatment. Furthermore, *INV*, *PGI*, *β-amylase*, *HXK*, and *SS* exhibited continuous downregulation, with their expression levels reduced in RNAi lines compared with WT and further significantly decreased after exogenous GA application (Figure 7B).

Integrating metabolomic and transcriptomic data shows that GA deficiency did not simply suppress primary metabolism but reorganized the sugar metabolic landscape in a bidirectional manner, with decreased glucose, trehalose, and inositol but increased galactose, mannose, and raffinose. Exogenous GA restored certain starch-related genes (*ISA*, *SBE*, *PGM*, *AGPase*), while sucrose metabolism genes (*SPS*, *FRK*, *SPP*) showed little change, and *INV*, *PGI*, *β-amylase, HXK,* and *SS* were further suppressed, indicating multilayered regulation by GA.

Collectively, these results indicate that sugar metabolism is closely associated with GA-mediated growth regulation, with exogenous supplementation partially restoring the metabolic state.

## 3. Discussion

**Figure 8:**
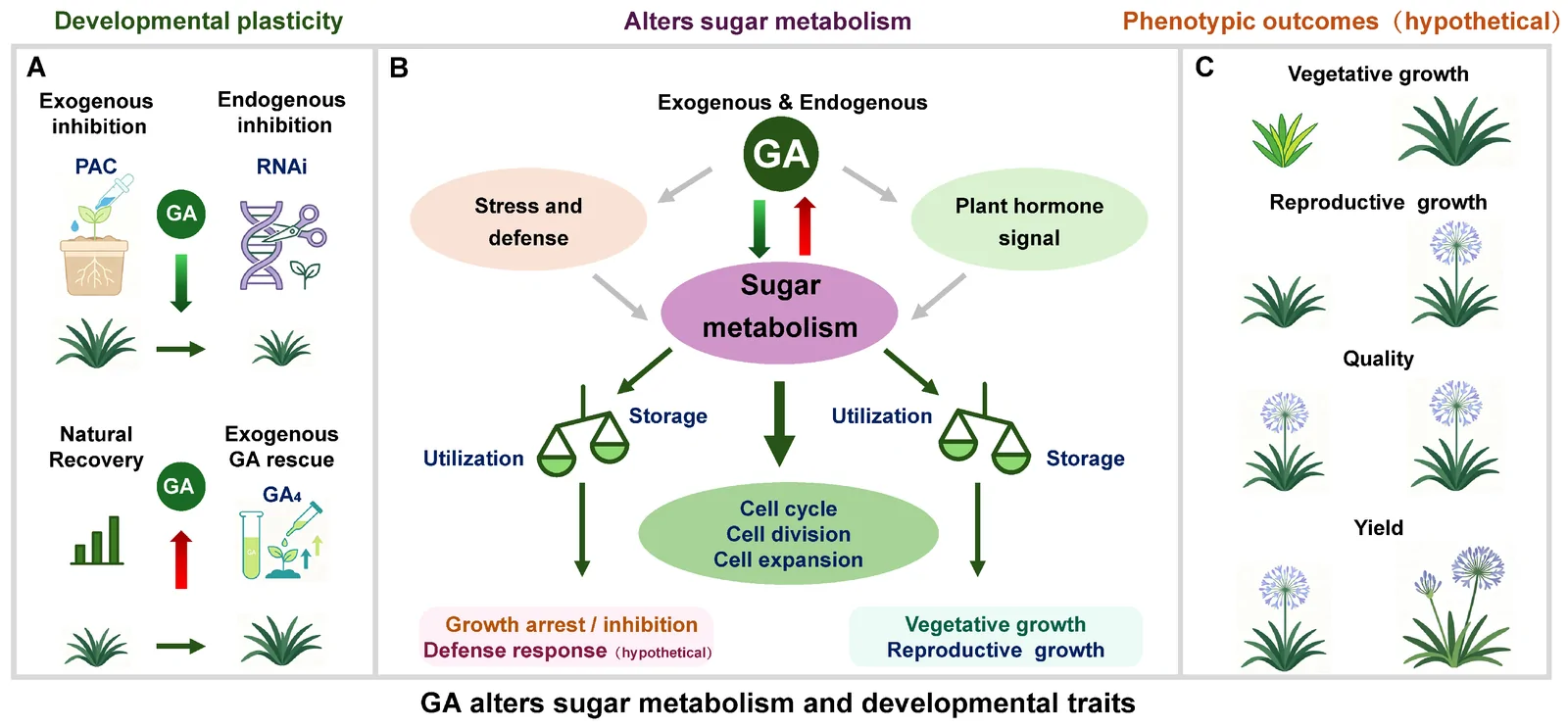
A working model illustrating how GA orchestrates sugar metabolism to mediate developmental plasticity and carbon partitioning in *A. praecox* subsp. *orientalis*. (**A**) This model is built upon three orthogonal perturbation strategies, including gradient PAC treatment, *GA20ox* RNAi silencing, and exogenous GA rescue, which collectively establish an integrative framework linking GA signaling to metabolic reprogramming and developmental outcomes. (**B**) GA globally reprograms primary carbon flux through multi-level regulation of the sugar metabolic network. This network serves as the major common pathway, translating upstream hormonal changes and stress responses into observable phenotypic outputs. GA promotes sugar utilization and growth, whereas GA deficiency redirects carbon toward storage and defense, thereby governing growth, source–sink allocation, and environmental adaptation. (**C**) GA functions dually as a developmental plasticity enabler, allowing plants to alter growth and reproductive programs in response to GA availability, and as a tunable hormonal switch, offering a precise entry point for agronomic manipulation. These findings provide a theoretical basis for precision manipulation of GA pathways to optimize plant architecture and carbon partitioning in ornamental and crop species.

The present study, combining gradient PAC treatments, GA20ox-RNAi transgenic lines, and exogenous GA rescue experiments in *A*. *praecox* subsp. *orientalis*, provides a characterization of the GA-mediated developmental and metabolic regulatory patterns in this ornamental species. Our results confirm and extend the established role of GA as a growth-promoting plant hormone [12,13], while offering species-specific insights into the dose-dependent control of carbohydrate metabolism and developmental plasticity. Our working model integrates three complementary lines of evidence (Figure 8): (1) convergent phenotypic and metabolic responses across three perturbation strategies; (2) identification of sugar metabolism as a major downstream component in which GA regulates growth and development; (3) demonstration of GA’s dual function as both a regulator of developmental plasticity and a modulator of source–sink dynamics affecting vegetative growth and reproductive transition [37].

### 3.1. GA as an Important Regulator of Metabolism and Development

Previous studies have established that GA promotes cell elongation and division primarily through the degradation of DELLA repressors [38,39,40,41,42]. Our multi-scale analyses, based on three independent perturbation strategies (Figure 8A), are consistent with this model and further demonstrate that the regulatory scope of GA in *A. praecox* encompasses reprogramming of sugar metabolism. Silencing of *GA20ox* led to a comprehensive reduction in glucose, accompanied by marked suppression of key sugar metabolic genes, including *sucrose synthase* (*SS*) and *β-amylase*. Notably, the transcriptional reprogramming of sugar metabolism genes exceeded that of hormone signaling or stress-responsive genes, suggesting that sugar metabolism constitutes a major effector layer of GA action in this species [43].

This observation is consistent with the view that GA acts not merely as a signaling molecule but also as a metabolic rheostat [21]. Under GA sufficiency, growth programs are associated with the activation of signaling cascades and the provision of energy and carbon skeletons essential for cell expansion [44]; conversely, under GA deficiency, impaired sugar metabolism correlates with restricted substrates for growth, providing a potential mechanistic basis for the dwarf phenotype [45]. The accumulation of DELLA proteins under low-GA conditions, which may directly affect key sugar metabolic enzymes, offers a plausible molecular explanation for this metabolic constraint [10,38,46,47]. This interpretation is consistent with previous reports in *Arabidopsis thaliana*, in which reduced GA levels led to DELLA stabilization and consequent promotion of sucrose-induced anthocyanin accumulation via upregulation of sucrose transporters and anthocyanin biosynthetic genes [48]. Thus, our findings recapitulate and extend the established GA–DELLA–sugar regulatory logic to an ornamental perennial species.

Furthermore, the downregulation of cell wall remodeling genes, such as *XET2*, under GA deficiency suggests that GA coordinates cell elongation through a dual mechanism: modulating sugar metabolism to provide carbon skeletons and energy [49] while simultaneously maintaining the expression of cell wall-modifying enzymes required for expansion [50,51]. Our results show that under significant suppression of *XET2* expression in RNAi plants, cell longitudinal axis shortened while width increased (Supplementary Figure S1), validating the classical model in which GA promotes elongation growth by inducing *XET2* expression. In addition, our RNAi data revealed that cytokinin (CTK) signaling pathways were significantly perturbed upon *GA20ox* silencing, which aligns with the coordinated GA-CTK regulation of floral development observed in kiwifruit, where both pathways act through DELLA and AHP components to fine-tune flowering time [52,53]. Similarly, elevated endogenous GA_3_ in strawberry is associated with activation of flowering genes and promotion of nutrient uptake [54]. Collectively, these observations confirm that the GA-DELLA module in *A. praecox* operates in a manner broadly analogous to that reported in model and crop species, while also revealing species-specific features in transcriptional responses. These findings establish GA as a tunable hormonal switch (Figure 8B): its manipulation not only elicits immediate metabolic and developmental responses but also reshapes carbon allocation dynamics across seasons, offering a precise entry point for agronomic intervention [55,56].

### 3.2. Dose-Dependent Effects and Phenotypic Plasticity

Gradient PAC treatments provide direct evidence for the dose-dependent nature of GA-mediated phenotypic plasticity [57,58]. Our results reveal that GA signaling is associated with qualitative shifts in developmental programs at discrete thresholds. Notably, 200 mg·L^−1^ PAC completely abolished floral development, converting floral primordia into vegetative leaf-like buds [59,60]. This threshold-driven transition corroborates the essential role of GA in maintaining floral meristem identity [5,52,61] and demonstrates that, when GA falls below a critical level, the developmental program switches from reproductive to vegetative growth [62,63]. This represents a dramatic example of developmental plasticity, enabling plants to prioritize survival over reproduction under extreme metabolic constraint [64].

This plasticity is also evident in carbon allocation dynamics. Plants dynamically adjust sugar partitioning in response to GA dose [28,65]; for example, graded GA-deficient tomato mutants exhibit dose-dependent carbon redistribution [66]. In our study, starch and soluble sugar content peaked at the highest PAC concentrations, revealing a relative surplus likely due to impaired utilization. These metabolic adjustments are consistent with active reallocation strategies, supporting the model in which GA deficiency is associated with a redirection of carbon toward storage (Figure 8B) [67].

Unlike annual herbaceous plants, *A. praecox* possesses prominent underground storage organs (rhizomes and fleshy roots) and exhibits two vegetative growth periods in the cultivated environment (March–May and September–November). According to the classical theory of assimilate partitioning, during the first year of exogenous treatment, growth inhibition may result in greater accumulation of assimilates in the tubers. Indeed, plants previously treated with high PAC concentrations not only recovered from dwarfism but also displayed significantly enhanced vegetative growth in the following year. This reversal from inhibition to stimulation suggests that growth restriction during the first year led to excessive accumulation of photoassimilates in vegetative organs, forming a carbon legacy that was remobilized to fuel compensatory growth once the PAC constraint was removed [68]. This redirection is consistent with our observed accumulation of starch and soluble sugars under PAC treatment—a metabolic signature of carbon surplus resulting from utilization blockade rather than enhanced supply. This mechanism is particularly pronounced in bulbous perennials, whose evolutionary strategy relies on seasonal carbon storage and retrieval to survive unfavorable periods and support rapid regrowth. Vegetative growth and reproductive development are interconnected, and floral transition and development are exceptionally carbon-demanding processes [62]; this likely explains why reproductive phenotypes showed more pronounced differences. In the RNAi experiment, GA biosynthesis deficiency resulted in severe failure of flowering, and this flowering phenotype was not restored by GA rescue, preliminarily suggesting that endogenous and exogenous GA regulation may operate through distinct modes in the control of reproductive traits.

### 3.3. Sugar Metabolism as a Major Downstream Component of GA-Mediated Regulation

Among the three perturbation strategies, the *GA20ox*-RNAi lines coupled with exogenous GA rescue provide the most direct test of our model: despite persistent blockade of endogenous GA biosynthesis, exogenous GA application achieved near-complete phenotypic recovery. Targeted metabolomics confirmed that exogenous GA restored bioactive GA levels to above WT levels, accompanied by increased catabolite GA_8_, indicating that the recovery is GA-dependent and that the sugar metabolic network in *A. praecox* retains sufficient responsiveness to exogenous hormone even when endogenous synthesis is compromised [28]. However, we acknowledge that isotope-labeled tracer experiments or detailed time-course analyses would be required to definitively distinguish between applied and endogenously synthesized GA_4_, and this represents an important direction for future studies.

KEGG enrichment analysis further pinpointed the starch and sucrose metabolism pathway as a significantly enriched category among GA-responsive genes. Within this pathway, the coordinated downregulation of *β-amylase* and upregulation of *α-amylase* under GA deficiency is consistent with a shift from utilization toward storage—a pattern that echoes the classic GA-induced amylase expression in barley aleurone [69].

PAC treatment alone does not specifically demonstrate GA-mediated effects, as paclobutrazol may affect other cytochrome P450-dependent processes. Notably, the GA-suppressing effect of PAC in *A. praecox* was confirmed in our previous study, which demonstrated that PAC significantly reduced endogenous GA content, especially GA_4_, in this species [36]. However, *GA20ox* silencing recapitulated the core PAC-induced phenotypes, and exogenous GA achieved near-complete phenotypic rescue, providing strong evidence that the observed effects are predominantly GA-dependent. The relationship between steady-state mRNA abundance and final phenotypic output is inherently indirect and multilayered. Many enzymes involved in carbohydrate metabolism—such as β-amylase, glucan–water dikinase, and enzymes controlling sucrose partitioning—are subject to post-translational regulation, including allosteric modulation, reversible phosphorylation, and protein turnover. The recovery of sugar metabolism upon GA rescue revealed a hierarchical regulatory logic: genes involved in starch biosynthesis (*SBE*, *PGM*, *AGPase*) were rapidly and fully restored by exogenous GA, suggesting they are direct or early targets of the GA-DELLA module [70]; in contrast, genes governing sucrose catabolism and *β-amylase* remained unrecovered or were further suppressed upon GA repletion, indicating they are subject to indirect, multilayered regulation [10,21,47,71]. Such layered control likely ensures that storage pathways respond rapidly to hormone availability, while utilization pathways are gated by additional metabolic or developmental checkpoints [72].

Regardless of the perturbation mode, including PAC inhibition, RNAi silencing, or exogenous GA rescue, the ultimate growth phenotype showed consistent associations with sugar metabolic status. Sugar metabolism thus appears to function as a major downstream component through which hormonal signals, stress responses, and transcriptional reprogramming are integrated in association with observable phenotypic outputs (Figure 8B). This interpretation is consistent with multiple lines of evidence from our study: sugar metabolism-related genes showed the most pronounced transcriptional changes; GA deficiency was associated with bidirectional alterations in sugar pools, decreasing glucose, trehalose, and inositol while increasing galactose, mannose, raffinose, and fructose [73]; and exogenous GA largely reversed these shifts [10,46]. These findings are in line with independent reports on *A. thaliana mvk-1* mutants [48] and *Hylocereus polyrhizus* [74]. In strawberry, *FvMAPK6* removes blocks to sugar and color production, with sucrose further boosting this pathway and suggesting a regulatory loop in sugar metabolism [75]. Taken together, these observations position sugar metabolism as a major downstream component of the GA regulatory network.

Beyond their roles as metabolic intermediates, sugars also function as signaling molecules that modulate diverse physiological processes in plants [76]. In our GA-deficient lines, we observed depletion of glucose, trehalose, and inositol, alongside accumulation of raffinose. Glucose signaling via the hexokinase pathway [77] links sugar availability to energy status; its depletion under GA deficiency may therefore signal low energy and contribute to growth arrest. Trehalose and its precursor T6P regulate sugar-to-growth coupling through SnRK1 [78], and their reduction reinforces a storage-over-utilization state. Accumulation of RFOs suggests carbon redirection toward protective pathways [79], while decreased inositol may reflect impaired membrane turnover and cell wall biosynthesis [80]. Together, these data indicate that GA deficiency reprograms both the metabolic and signaling dimensions of the sugar pool. Whether these associations reflect direct causation or correlative readouts requires further validation through sugar feeding or genetic manipulation of key enzymes.

Synthesizing these observations, the GA–sugar metabolic axis appears to be associated with discrete phenotypic states [70]. Under sufficient GA signaling, balanced carbon flux is associated with normal growth and reproductive transition, while under GA deficiency, including PAC or RNAi treatments, impaired sugar utilization is associated with a redirection of carbon toward storage, correlating with dwarfism and reproductive arrest. Upon exogenous GA rescue, near-complete phenotypic recovery ensues. The reversibility of these transitions indicates that the dwarf phenotype is not a permanent structural defect but a reversible metabolic arrest, a finding with potential implications for precision management of GA pathways in ornamental production. We acknowledge that our conclusions rely mainly on transcriptomic and metabolomic data. Protein-level validation and flux analysis were not performed and will be needed to establish causality between GA perturbation, sugar metabolic flux, and phenotypic outcomes.

### 3.4. Implications for Ornamental Crop Management

From an applied perspective, our findings demonstrate that artificial GA manipulation can regulate desired traits through remodeling of the metabolic network. Figure 8C provides a conceptual framework in which modulating GA flux enables artificial control of vegetative growth and reproductive development. For example, through targeted GA manipulation, one can either promote or suppress vegetative growth, regulate reproductive development (including floral bud differentiation and flowering time control), and modulate the size and number of floral organs (via tillering). For ornamental crops such as *A. praecox*, this framework offers practical guidance for optimizing crop management through targeted GA regulation. The PAC concentrations used in this study (50–200 mg·L^−1^) are within the range commonly applied in ornamental horticulture for height control and plant architecture regulation. These doses effectively suppressed excessive vegetative growth while avoiding the severe phytotoxic effects observed at higher concentrations, supporting the practical relevance of our findings for optimizing PAC application strategies in *A. praecox* and related ornamental crops.

Importantly, the interplay between GA and sugar metabolism defines the operational boundaries of developmental plasticity and reveals an exploitable window for agronomic intervention. Future cultivation strategies should account for the systemic effects of GA on source–sink relationships, potentially harnessing carry-over cycles to optimize carbon partitioning for desired traits. The dual recognition of developmental plasticity as an intrinsic plant property and GA tunability as an agronomic opportunity offers a unified perspective for the rational manipulation of plant architecture and resource allocation [81].

## 4. Materials and Methods

### 4.1. Plant Materials and Growth Conditions

Four-year-old plants of *A. praecox* subsp. *orientalis* were grown in the greenhouse of Xinyang Agriculture and Forestry University, Xinyang, China (32°9′ N, 114°7′ E), under natural light conditions with indoor temperatures of approximately 20–25 °C (night) and 25–35 °C (day), a 14 h photoperiod maintained by timed shading and supplementary lighting, and 60–70% relative humidity. The plants were grown in pots (30 cm in diameter and 35 cm in depth) filled with a mixture of vermiculite/peat (1:1, *v*/*v*). All plants were subjected to routine management.

### 4.2. PAC Gradient Treatment and Phenotypic and Physiological Measurement

To evaluate the dose-dependent effects of PAC, uniformly growing *A. praecox* subsp. *orientalis* plants were treated with PAC solutions at concentrations of 0, 50, 100, 150, and 200 mg·L^−1^, applied as a soil drench at a volume of 500 mL per pot. Each treatment consisted of three biological replicates, with five plants per replicate. For phenotypic measurements, all five plants within each replicate were measured individually, and the mean value per replicate was used as the experimental unit for statistical analysis (*n* = 3 per treatment). Plants were arranged in a completely randomized design and re-randomized weekly to minimize positional effects. The treatment began in mid-May 2022 and was applied once every 90 days for a total of four applications (May, August, November, and February). Upon completion of the treatment, the following morphological parameters were measured in May 2023: plant height, tiller number, leaf number, leaf length, leaf width, inflorescence diameter, number of flowers per inflorescence, and pedicel length. Meanwhile, pedicel and scape samples were collected for paraffin sectioning and staining. Relative chlorophyll content (SPAD value) was determined using a SPAD-502 chlorophyll meter (Konica Minolta, Chiyoda, Japan), with three fully expanded leaves measured per plant and averaged. For physiological measurements, pedicels were used as the experimental material. Soluble sugar, starch, and soluble protein contents were measured by the anthrone colorimetric, iodine colorimetric, and Coomassie Brilliant Blue G-250 methods, respectively. After the treatment, all plants were maintained under the same conditions without further PAC application, and morphological parameters were re-measured in May 2024 to evaluate the carry-over effects of PAC treatment. All data were collected at the flower bud cracking stage (the day the floral bud first opens).

### 4.3. Paraffin Sectioning and Histochemical Staining

Fresh pedicel and scape samples were immediately fixed in FAA fixative (formalin/glacial acetic acid/70% ethanol = 5:5:90, *v*/*v*/*v*), dehydrated through a graded ethanol series, cleared with xylene, and embedded in paraffin. Sections were cut to a thickness of 8–10 μm using a rotary microtome (Leica RM2235, Nußloch, Germany). Slides were stained with hematoxylin (BBI, Sangon Biotech, Shanghai, China) for cell size observation and with periodic acid-Schiff (PAS) for polysaccharide identification. Morphological images of pedicel were captured using an Axio Scope A1 microscope (Carl Zeiss, Oberkochen, Germany).

### 4.4. Construction of GA20ox RNAi Transgenic Lines

A specific fragment of the *GA20ox* gene was amplified and inserted into the pTCK303-RNAi vector in both forward and reverse orientations. The construct was transformed into *Agrobacterium tumefaciens* EHA105 and introduced into embryogenic calli. Transgenic plants were selected on hygromycin (50 mg·L^−1^) and confirmed by PCR, and RNAi plants, obtained via somatic embryogenesis, were used as three biological replicates. Phenotypic traits were measured individually, and tissues were pooled for molecular analyses. WT plants served as controls. qRT-PCR confirmed significant downregulation of *GA20ox* in RNAi lines (*p* < 0.01).

### 4.5. Exogenous GA Rescue Experiment

Uniformly growing RNAi plants in the same transgenic line were selected for exogenous GA rescue treatment starting in May 2025, with three independent plants used as biological replicates. Exogenous GA_4_ (Sigma-Aldrich, St. Louis, MO, USA) was dissolved in a small volume of ethanol and diluted with distilled water to 50 mg·L^−1^ (containing 0.05% Tween-20). The 50 mL solution was sprayed once every 30 days for a total of four applications (RNAi_GA), with each RNAi line (RNAi) and wild-type (WT) plant sprayed with an equal volume of solvent (0.05% Tween-20) as controls. Leaf samples of WT, RNAi, and RNAi_GA were collected in December 2025; immediately before harvest, leaves were rinsed three times with distilled water containing 0.1% Tween-20, rinsed three times with only distilled water, and blotted dry. All metabolomic, transcriptomic, and hormonal analyses were performed on the youngest fully expanded leaf (middle age). Samples were then frozen in liquid nitrogen and stored at −80 °C for transcriptomic, metabolomic, and qRT-PCR analyses.

### 4.6. Transcriptome Sequencing and Differential Expression Analysis

Total RNA was extracted from leaf samples of WT, RNAi, and RNAi_GA groups using the RNAiso Plus kit (TaKaRa), and RNA quality was assessed by agarose gel electrophoresis and NanoDrop spectrophotometry. cDNA libraries were constructed and sequenced on an Illumina HiSeq 2500 platform (paired-end, 150 bp), and raw reads were filtered to remove adapters and low-quality reads. Since no reference genome is available for *A. praecox* subsp. *orientalis*, clean reads were assembled de novo using Trinity (v2.15.1) and clustered using Corset to obtain unigenes. Assembly completeness was assessed using BUSCO (v5.2.2) against the database. Quantification was performed using Bowtie2 (v2.3.5.1) and Ballgown, with expression levels calculated as FPKM.

Functional annotation was performed by BLASTX against NR, Swiss-Prot, and TAIR10; GO terms were assigned via Blast2GO (v5.2), and KEGG annotation via KAAS. Differential expression analysis was performed using DESeq2 (v1.34.0) in R (v4.1.2). Reads were normalized using the median-of-ratios method, and DEGs—defined as |log_2_FC| ≥ 1 and *P*_adj_ < 0.05—were identified using a negative binomial GLM with the Wald test, with dispersion estimates via empirical Bayes shrinkage. PCA and sample correlation heatmaps were generated to assess replicate quality.

### 4.7. Targeted Hormone Metabolomics Analysis

To profile the hormonal differences among WT, RNAi, and RNAi_GA groups, targeted plant hormone metabolomics was performed using LC-MS/MS. Leaf samples were ground in liquid nitrogen and extracted with methanol/water (7:3, *v*/*v*) containing an internal standard (L-2-chlorophenylalanine, 4 μg·mL^−1^), assisted by ultrasonic extraction. After centrifugation, the supernatant was filtered through a 0.22 μm membrane. Analysis was performed on a Waters ACQUITY UPLC system coupled with a Xevo TQ-S mass spectrometer (Waters Corporation, Milford, MA, USA), using an ACQUITY UPLC BEH C18 column (1.7 μm, 2.1 × 100 mm). Mobile phase A was 0.01% formic acid in water, and mobile phase B was 0.01% formic acid in acetonitrile, with gradient elution. Data were acquired in multiple reaction monitoring (MRM) mode and quantified by the internal standard method, with hormone contents expressed as ng·g^−1^ fresh weight. Pooled quality control (QC) samples were prepared by combining equal aliquots of all samples and injected every 10 injections to monitor instrument performance. Metabolite identification was performed at confidence Level 1 according to Metabolomics Standards Initiative (MSI) guidelines. All calibration parameters: RT, Equation, r, Weighting, LLOQ and ULOQ for each plant hormone compound are provided in Supplementary Table S6.

Raw peak intensities were normalized using internal standards. Univariate analysis used one-way ANOVA followed by Duncan’s multiple range test (*p* < 0.05), and multivariate analysis (PCA and OPLS-DA) was performed using SIMCA 14.1 with 7-fold cross-validation and 200 permutation tests. Differential metabolites were selected by VIP ≥ 1, *p* < 0.05, and |fold change| ≥ 1.0. Benjamini–Hochberg correction was applied for multiple testing, and data were log_2_-transformed and Pareto-scaled. All analyses were conducted using MetaboAnalyst 6.0.

### 4.8. Targeted Sugar Metabolomics Analysis

Targeted sugar metabolomics was performed using GC-MS/MS. Leaf samples were ground in liquid nitrogen and extracted with 70% methanol containing an internal standard (L-2-chlorophenylalanine, 4 μg·mL^−1^), assisted by ultrasonic extraction. After centrifugation, the supernatant was dried under a nitrogen stream, and the dried residue was derivatized by oximation with methoxyamine hydrochloride at 37 °C for 90 min, followed by silylation with BSTFA (containing 1% TMCS) at 70 °C for 60 min. Analysis was performed on an Agilent 7890B GC system coupled with a 5977A mass spectrometer (Agilent Technologies, Santa Clara, CA, USA), using a DB-5MS fused-silica capillary column (30 m × 0.25 mm × 0.25 μm). Data were acquired in full-scan mode (*m*/*z* 50–500). Metabolites were identified based on the NIST and Fiehn databases, with identification confidence at Level 2 (putative identification based on spectral matching) according to MSI guidelines. All calibration parameters, including linear ranges, R^2^ values, LOD, and LOQ for each sugar compound, are provided in Supplementary Table S7. A total of 20 sugar compounds were quantified. Pooled QC samples were injected every 10 injections to monitor instrument stability. DAMs were selected with the same criteria as in Section 4.7.

### 4.9. Targeted GA Metabolite Quantification

To evaluate changes in endogenous GA biosynthetic pathways among WT, RNAi, and RNAi_GA groups, targeted GA quantification was performed by UPLC-MS/MS. Leaf samples were collected, frozen in liquid nitrogen, and ground into fine powder. Briefly, 50 mg of powdered tissue was extracted with ice-cold ethyl acetate/methanol/formic acid (80:15:5, *v*/*v*/*v*) containing isotope-labelled external and internal standards (Supplementary Tables S8 and S9), samples were vortexed and ultrasonically extracted on ice, the extracts were centrifuged at 12,000 rpm for 15 min at 4 °C, and supernatants were purified using Oasis HLB solid-phase extraction cartridges (Waters, Milford, MA, USA). Purified samples were reconstituted for UPLC-MS/MS detection.

Chromatographic separation was performed on a Waters ACQUITY BEH C18 column (2.1 × 100 mm, 1.7 μm) with a Waters ACQUITY UPLC system. The mobile phase comprised 0.05% aqueous formic acid (phase A) and methanol (phase B) with gradient elution. Column temperature was 40 °C with a flow rate of 0.35 mL·min^−1^. Mass spectrometric detection was performed on a Waters Xevo TQ-S triple quadrupole mass spectrometer in ESI negative mode with MRM, and absolute quantification was achieved using the isotope-labelled internal standard method, with calibration curves ranging from 0.1 to 100 ng·mL^−1^ (R^2^ > 0.99 for all analytes). Extraction recoveries, R value, LOD, and LOQ for each GA are reported in Supplementary Table S10. Pooled QC samples were injected every 10 injections, with RSD < 15% for peak area and < 5% for retention time. Metabolite identification was at MSI Level 1. Raw peak areas were normalized to the corresponding isotope-labelled internal standard, and final concentrations were calculated based on sample fresh weight.

### 4.10. Quantitative Real-Time PCR (qRT-PCR) Validation

One microgram of total RNA was used for reverse transcription with the PrimeScript™ 1st Strand cDNA Synthesis Kit (TaKaRa, Dalian, China). Based on the transcriptome results, key DEGs involved in hormone signaling, oxidative stress, and sugar metabolism pathways were selected for qRT-PCR validation using an ABI 7500 Fast PCR Detection System (Life Technologies, Carlsbad, CA, USA) according to the manufacturer’s instructions. The expression values were normalized against *β-actin* as the internal reference gene, and relative expression levels were calculated using the 2^−ΔΔCt^ method [82]. Primer sequences are listed in Supplementary Table S11. Each sample had three technical replicates, and the results were visualized as log_2_ FC heatmaps using the Metware Cloud online platform (https://cloud.metware.cn).

### 4.11. Statistical Analysis

All data are presented as mean ± standard error (SE). For the PAC experiment, the replicate mean (*n* = 3) was used; for the transgenic experiment, individual line values (n = 3) were used. Statistical analyses were performed using one-way analysis of variance (ANOVA) followed by Duncan’s multiple range test (*p* < 0.05) using SPSS v20.0 (SPSS Inc., Chicago, IL, USA). For transcriptomic data, differential expression analysis was based on FDR correction, with *p* < 0.05 considered significant.

## 5. Conclusions

In summary, this study identifies GA as an important regulator of the GA–sugar metabolic axis, supporting and extending its established role beyond growth promotion. GA is associated with the regulation of growth, source–sink allocation, and stress adaptation through effects on carbon partitioning, and its effects are dose-dependent, with critical thresholds associated with qualitative developmental shifts that are correlated with developmental plasticity. Overall, plant phenotypes appear to emerge from the integration of GA signaling and sugar metabolism, reflecting a flexible and adaptive regulatory system.

## Data Availability

Raw sequence data reported in this paper have been deposited in the Genome Sequence Archive (GSA) in the BIG Data Center, Chinese Academy of Sciences under accession code CRA048152 that are publicly accessible at http://bigd.big.ac.cn/gsa (accessed on 9 July 2026).

## Supplementary Materials

The following supporting information can be downloaded at: https://www.mdpi.com/article/10.3390/plants15172699/s1, Figure S1: Microscopic observation of epidermal leaves cells; Figure S2: *GA20ox* silencing (RNAi) caused dwarfism and flowering failure; Figure S3: Principal component analysis (PCA) model of transcriptomics; Figure S4: Correlation analysis among samples of RNA-Seq; Figure S5: Numbers of DEGs in different comparisons; Figure S6: The expression analysis of transcripts based on RNA-Seq and qRT-PCR; Figure S7: Numerical expression values of DEGs conducted by qRT-PCR; Table S1: Plant hormone targeted metabolomics data; Table S2: Gibberellin targeted metabolomics data; Table S3: Basic information of RNA-Seq; Table S4: De novo assembly information of RNA-Seq; Table S5: Soluble sugar targeted metabolomics data; Table S6: Calibration parameters of plant hormone targeted metabolomics; Table S7: Calibration parameters of soluble sugar targeted metabolomics; Table S8: Information of reference external standard; Table S9: Information of reference internal standard; Table S10: Calibration parameters of GA targeted metabolomics; Table S11: Primer sequences used for qRT-PCR.

## Abbreviations

The following abbreviations are used in this manuscript:

α-amylase	alpha-amylase
β-amylase	beta-amylase
AGL9	agamous-like 9
AGPase	ADP-glucose pyrophosphorylase
APX	ascorbate peroxidase
ARF1	auxin response factor 1
ARF7	auxin response factor 7
AUX/IAA	auxin/indole-3-acetic acid
AUX22B	auxin-responsive protein 22b
BRH1	brassinosteroid-responsive gene 1
BRI1	brassinosteroid-insensitive 1
CAT	catalase
CDK	cyclin-dependent kinase
CDPK	calcium-dependent protein kinase
CYCLIN/SDS	cyclin and SDS protein
CYCD3	cyclin D3
DREB	dehydration-responsive element-binding protein
ERF2	ethylene response factor 2
ETR2	ethylene receptor 2
FRK	fructokinase
GA	gibberellin
GAI	gibberellin-insensitive (DELLA protein)
GA20ox	gibberellin 20-oxidase
GH3	Gretchen Hagen 3
GID1	gibberellin-insensitive dwarf 1
GID1a	gibberellin-insensitive dwarf 1a
GO	Gene Ontology
GPX	glutathione peroxidase
GR	glutathione reductase
GWD	glucan water dikinase
HXK	hexokinase
IAA13	indole-3-acetic acid-induced protein 13
IAA20	indole-3-acetic acid-induced protein 20
INV	invertase
ISA	isoamylase
KEGG	Kyoto Encyclopedia of Genes and Genomes
MAPK	mitogen-activated protein kinase
MET1	DNA methyltransferase 1
PGI	phosphoglucose isomerase
PGM	phosphoglucomutase
PIN1	PIN-formed 1
PIN3A	PIN-formed 3A
POD	peroxidase
PYL4	pyrabactin resistance 1-like 4
qRT-PCR	quantitative real-time polymerase chain reaction
SAUR76	small auxin-up RNA 76
SBE	starch branching enzyme
SnRK1	sucrose non-fermenting 1-related protein kinase 1
SOD	superoxide dismutase
SODC	superoxide dismutase (Cu/Zn)
SPS	sucrose phosphate synthase
SPP	Sucrose-6-phosphate phosphatase
SS	starch synthase
SUS	sucrose synthase
UGPase	UDP-glucose pyrophosphorylase
UXS	UDP-xylose synthase
WOX9	WUSCHEL-related homeobox 9
XET2	xyloglucan endotransglucosylase/hydrolase 2
YUCCA4	flavin-binding monooxygenase family protein
