# Supplementary material for: Gibberellin Homeostasis Perturbation Alters Sugar Metabolism and Developmental Traits in Agapanthus praecox subsp. orientalis
Source: Plants (Basel). 2026 Sep 2;15(17):2699. doi: 10.3390/plants15172699 (PMC13567497; doi:10.3390/plants15172699)
Supplement: Supplementary file 1 [file plants-15-02699-s001.zip › Supplementary Figures.pdf]

Supplementary Figures

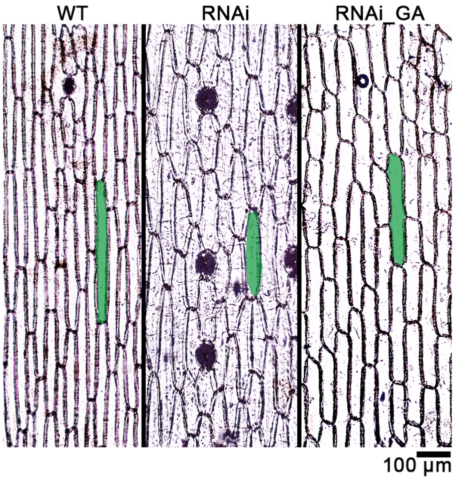

Figure S1. Microscopic observation of epidermal leaves cells. bar = 200 µm.

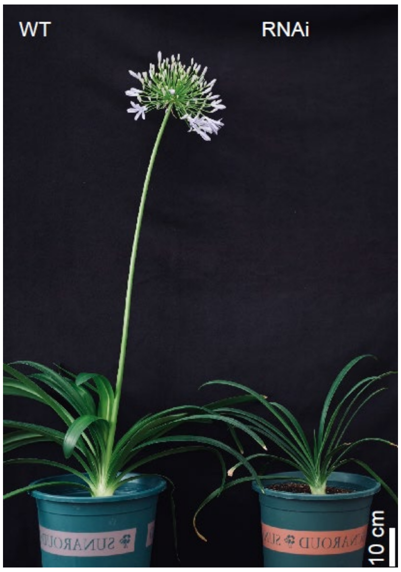

Figure S2. *GA20ox* silencing (RNAi) caused dwarfism and flowering failure; wild-type (WT) and RNAi line (RNAi), bar =10 cm.

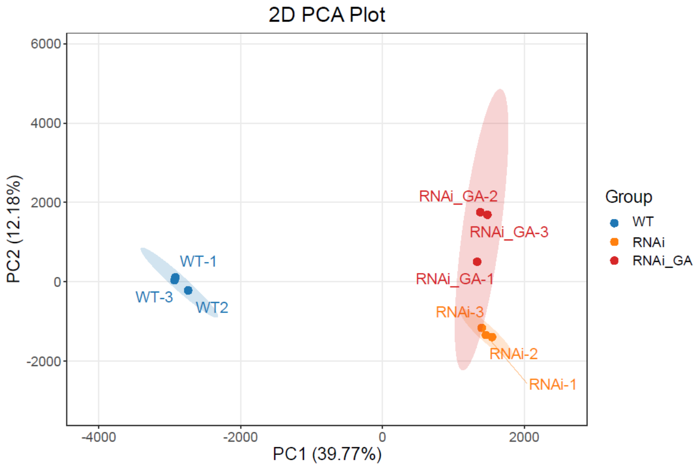

Figure S3. Principal component analysis (PCA) model of transcriptomics.

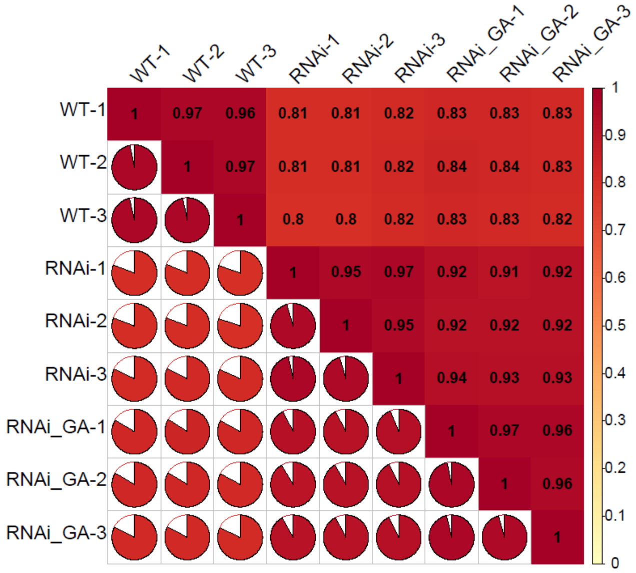

Figure S4. Correlation analysis among samples of RNA-Seq; the horizontal axis represents the sample clusters, and colors from yellow to red indicate the correlation index from low to high.

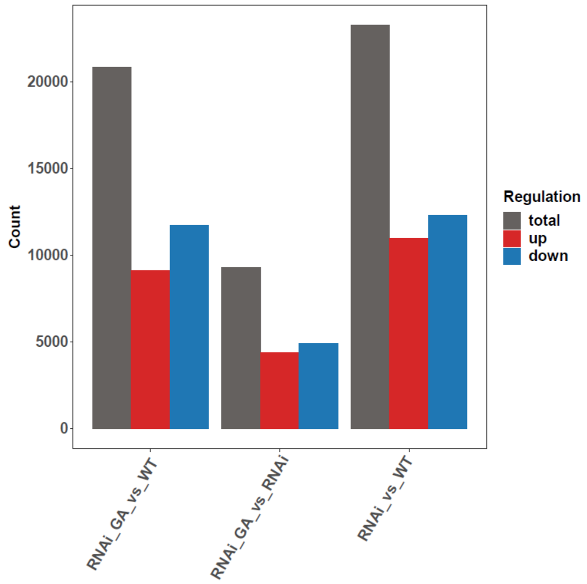

Figure S5. Numbers of DEGs in different comparisons.

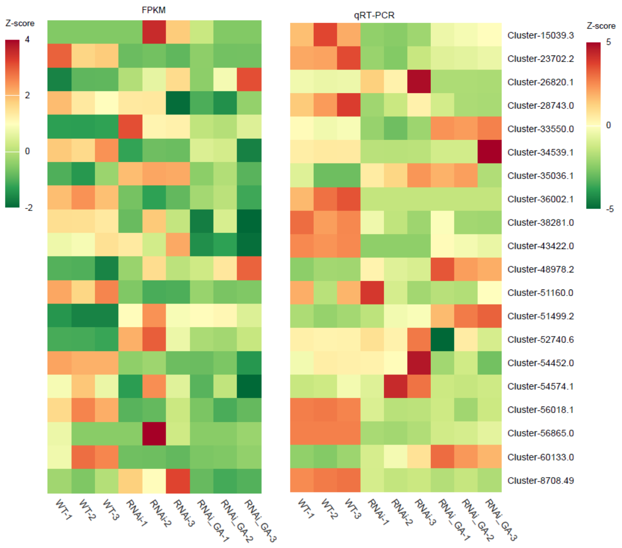

Figure S6. The expression analysis of transcripts based on RNA-Seq and qRT-PCR. Colors from green to red indicate transcripts expression levels from low to high, and values are log_2-fold change.

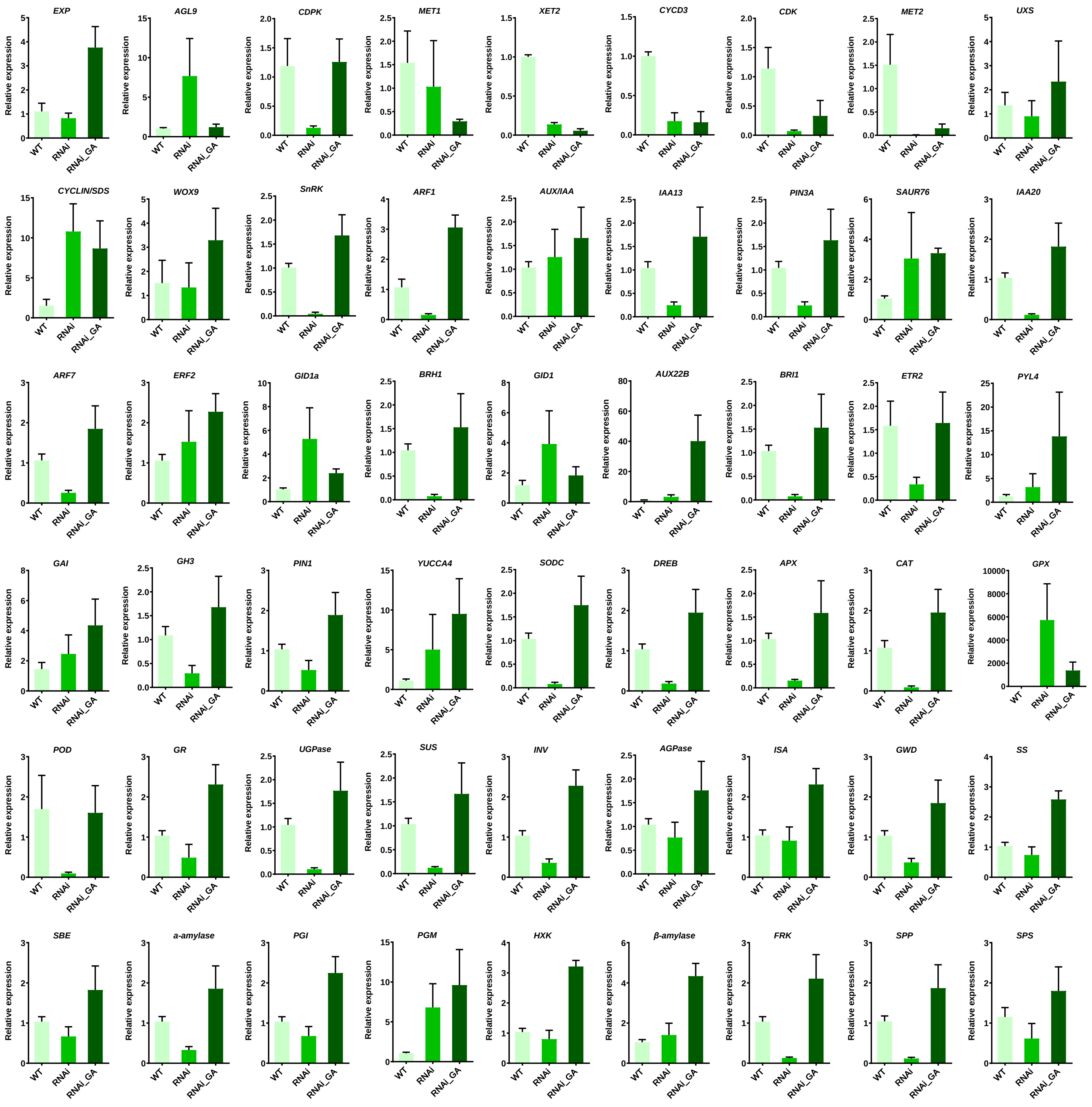

Figure S7. Numerical expression values of DEGs conducted by qRT-PCR. Data are presented as mean ± SE; n = 3.
