# Supplementary material for: Gibberellin Homeostasis Perturbation Alters Sugar Metabolism and Developmental Traits in Agapanthus praecox subsp. orientalis
Source: Plants (Basel). 2026 Sep 2;15(17):2699. doi: 10.3390/plants15172699 (PMC13567497; doi:10.3390/plants15172699)
Supplement: Supplementary file 1 [file plants-15-02699-s001.zip › Supplementary Table S1.pdf]

Table S1: The targeted plant hormone metabolomics

Index	Class	Formula	CK-1	CK-2	CK-3	RNAi-1	RNAi-2	RNAi-3	RNAi_GA-1	RNAi_GA-2	RNAi_GA-3
ABA	ABA	$C_{15}H_{20}O_4$	39.8236196	43.8945412	34.6902591	27.9920031	34.3949412	30.0963336	12.4609407	14.8009699	13.7417128
TRP	Auxin	$C_{11}H_{12}N_2O_2$	2641.8456	3122.62767	2520.44146	12488.1266	14538.3081	13392.501	8428.02722	9581.60135	7339.52602
2MeScZR	CK	$C_{16}H_{23}N_5O_5S$	1.05946063	1.04436754	0.897629559	1.35303784	1.92261492	1.62630089	2.60049269	2.71758709	2.40575401
DHZ7G	CK	$C_{16}H_{25}N_5O_6$	0.215437117	0.288182352	0.220710173	0.484259801	0.607126364	0.520642916	0.344496648	0.366862134	0.356144864
DHZROG	CK	$C_{21}H_{33}N_5O_{10}$	0.5986682	0.680209352	0.60515595	0.640479325	0.902634341	0.76002285	0.292525904	0.264773852	0.219189096
cZR	CK	$C_{15}H_{21}N_5O_5$	1.57412065	2.14884807	1.62042466	3.50938658	4.73989819	4.09752804	5.41637038	6.25870942	5.09449832
tZ	CK	$C_{10}H_{13}N_5O$	0.446232106	0.465053316	0.399263436	0.52252048	0.576831444	0.562746676	0.435254978	0.458382819	0.466584207
tZ9G	CK	$C_{16}H_{23}N_5O_6$	1.89251022	1.95195167	1.40956814	8.08525941	11.7430468	10.080053	5.82550284	5.45246932	5.92083911
BAP7G	CK	$C_{18}H_{21}N_5O_5$	2.06299335	1.97585111	1.67190259	1.55977667	1.81004146	1.60604227	1.00438846	1.07321605	0.920158817
IAA-Asp	Auxin	$C_{14}H_{14}N_2O_5$	0.721694785	0.687512229	0.613913148	1.90625853	1.81788151	2.04446926	1.56614689	2.10528998	2.21311844
IPR	CK	$C_{15}H_{21}N_5O_4$	1.21714213	1.29691107	1.02853647	2.38388678	3.06108312	2.29067304	2.44236591	2.8248466	2.55875223
ACC	ETH	$C_4H_7NO_2$	69.4220348	77.5738603	70.9335413	66.4160328	78.9609572	61.8846074	60.1777733	67.3552553	59.0164259
GA_{24}	GA	$C_{20}H_{26}O_5$	7.06472393	5.57151242	7.60427063	13.6868295	17.8420707	14.926075	4.69044596	4.97938935	4.99824362
GA_{29}	GA	$C_{19}H_{24}O_6$	612.5409	614.684015	610.506238	1434.88882	2047.31318	1520.74159	1875.54094	2098.74555	2662.05719
GA_9	GA	$C_{19}H_{24}O_4$	5.28637526	4.50701917	4.3600048	2.48193388	2.3371589	2.55859213	2.77095185	2.10385491	2.11275975
JA	JA	$C_{12}H_{18}O_3$	301.262781	345.61485	357.231286	453.179247	444.321998	456.114977	310.272755	298.436263	313.996636
JA-ILE	JA	$C_{18}H_{29}NO_4$	282.978016	372.136079	395.038388	142.000683	178.022932	149.87069	271.350569	287.504949	215.519493
SA	SA	$C_7H_6O_3$	5.8577454	8.17386519	6.49973608	9.02116247	7.45185243	7.86887204	8.43590004	6.26472189	8.68202058
Phe	SA	$C_9H_{11}NO_2$	3138.16462	3637.83995	3470.3215	4765.50614	5743.12552	5344.80162	3449.08066	3789.09343	3497.79834
tZOG	CK	$C_{16}H_{23}N_5O_6$	4.08908487	4.81510468	3.29822457	10.4459235	13.022224	12.1048504	7.30729378	7.61992775	7.5766129
IAA	Auxin	$C_{10}H_9NO_2$	8.20986708	9.59540697	7.70328695	12.3027599	17.7407641	13.0841556	12.1063592	8.96736441	9.9052543
iPRMP	CK	$C_{15}H_{22}N_5O_7P$	4.37080777	4.71326551	4.90391075	9.19058416	12.2842412	10.8980837	6.92205912	6.86601841	7.51590639
IAN	Auxin	$C_{10}H_8N_2$	0.885201943	1.02827235	1.19753839	1.17440511	1.0047754	1.30209285	1.41432345	1.07013806	1.26810063

JA-Val	JA	$C_{17}H_{27}NO_4$	3.03888037	3.69991195	3.56952975	N/A	N/A	N/A	10.724426	11.6900238	8.02382248
tZRMP	CK	$C_{15}H_{22}N_5O_8P$	2.76339468	3.5322344	3.10513436	2.17672372	3.20161629	2.41295181	N/A	N/A	N/A
GA_{34}	GA	$C_{19}H_{24}O_6$	8.2648773	6.65297887	7.99620921	15.4143505	24.9688287	14.997715	N/A	N/A	N/A
cZRMP	CK	$C_{15}H_{22}N_5O_8P$	N/A	N/A	N/A	1.25049737	1.56166037	1.25510231	1.33779206	1.42338183	1.3755096
iP9G	CK	$C_{16}H_{23}N_5O_5$	1.29114775	1.12806202	1.31945058	N/A	N/A	N/A	1.79312017	1.4646254	1.84515882
JA-Phe	JA	$C_{21}H_{27}NO_4$	0.405352761	0.363130992	0.509923225	0.523759021	0.408312343	0.421946406	N/A	N/A	N/A
OPDA	JA	$C_{18}H_{28}O_3$	12.1051892	17.2864899	14.1627159	112.607519	138.718251	110.084649	N/A	N/A	N/A
ICAld	Auxin	C_9H_7NO	3.52868098	4.73975249	4.65878119	9.47847182	13.7525451	7.52061695	N/A	N/A	N/A
IAA-Ala	Auxin	$C_{13}H_{14}N_2O_3$	N/A	N/A	N/A	1.07457578	1.36816488	0.911521084	N/A	N/A	N/A
cZ	CK	$C_{10}H_{13}N_5O$	N/A	N/A	N/A	N/A	N/A	N/A	0.32639679	0.436218329	0.327822581
GA_{19}	GA	$C_{20}H_{26}O_6$	2.09066207	3.03717472	2.55283109	N/A	N/A	N/A	N/A	N/A	N/A
GA_3	GA	$C_{19}H_{22}O_6$	N/A	N/A	N/A	N/A	N/A	N/A	8.02425335	11.4296071	10.1201267
tZR	CK	$C_{15}H_{21}N_5O_5$	1.45828988	1.28835111	1.14327255	N/A	N/A	N/A	N/A	N/A	N/A
KR	CK	$C_{15}H_{17}N_5O_5$	0.258343558	0.366051164	0.305614203	N/A	N/A	N/A	N/A	N/A	N/A
cZROG	CK	$C_{21}H_{31}N_5O_{10}$	N/A	N/A	N/A	3.5568071	4.27453296	4.38873598	N/A	N/A	N/A
GA_7	GA	$C_{19}H_{22}O_5$	N/A	N/A	N/A	N/A	N/A	N/A	35.5670967	23.1167854	29.3724025
IP	CK	$C_{10}H_{13}N_5$	0.0991666667	0.114114166	0.0758901152	N/A	N/A	N/A	N/A	N/A	N/A
MEJA	JA	$C_{13}H_{20}O_3$	3.9081544	3.13045392	3.27519194	N/A	N/A	N/A	N/A	N/A	N/A
SAG	SA	$C_{13}H_{16}O_8$	10.5645961	6.79517218	8.26621881	N/A	N/A	N/A	N/A	N/A	N/A
JA-ACC	JA	$C_{16}H_{22}NO_4^-$	1.07542689	1.52069556	1.35115403	N/A	N/A	N/A	N/A	N/A	N/A
OPC-4	JA	$C_{14}H_{22}O_3$	27.6776585	31.7308257	33.5597409	N/A	N/A	N/A	N/A	N/A	N/A
