# Supplementary material for: Gibberellin Homeostasis Perturbation Alters Sugar Metabolism and Developmental Traits in Agapanthus praecox subsp. orientalis
Source: Plants (Basel). 2026 Sep 2;15(17):2699. doi: 10.3390/plants15172699 (PMC13567497; doi:10.3390/plants15172699)
Supplement: Supplementary file 1 [file plants-15-02699-s001.zip › Supplementary Table S10.pdf]

**Table S10: Calibration parameters of GA targeted metabolomics**

| Index | Class | RT | Equation | r | Weighting | LLOQ | ULOQ |
|---|---|---|---|---|---|---|---|
| $GA_{12}$-ald | gibberellin | 10.76 | y = 0.89601 x - 0.02241 | 0.99406 | 1 / x^2 | 0.2 | 100 |
| $GA_8$ | gibberellin | 3.47 | y = 7.06395 x + 0.08599 | 0.99665 | 1 / x^2 | 0.01 | 10 |
| $GA_{29}$ | gibberellin | 3.70 | y = 0.32123 x + 0.01413 | 0.99335 | 1 / x^2 | 0.02 | 10 |
| $GA_{19}$ | gibberellin | 4.13 | y = 0.21366 x - 0.00615 | 0.99558 | 1 / x | 0.1 | 100 |
| $GA_3$ | gibberellin | 4.18 | y = 1.80253 x + 0.00484 | 0.99321 | 1 / x^2 | 0.02 | 10 |
| $GA_1$ | gibberellin | 4.24 | y = 1.76519 x + 0.01825 | 0.99262 | 1 / x^2 | 0.02 | 10 |
| $GA_6$ | gibberellin | 4.78 | y = 1.24327 x + 0.00462 | 0.99158 | 1 / x^2 | 0.02 | 10 |
| $GA_{24}$ | gibberellin | 5.56 | y = 0.85218 x - 0.07596 | 0.99687 | 1 / x | 0.1 | 100 |
| $GA_5$ | gibberellin | 5.89 | y = 1.20430 x + 0.01411 | 0.99028 | 1 / x^2 | 0.01 | 10 |
| $GA_{20}$ | gibberellin | 6.05 | y = 1.83024 x + 0.00483 | 0.99968 | 1 / x | 0.02 | 10 |
| $GA_{51}$ | gibberellin | 6.33 | y = 1.99331 x + 1.65161e-4 | 0.99223 | 1 / x^2 | 0.02 | 10 |
| $GA_{34}$ | gibberellin | 6.37 | y = 10.70010 x + 0.21612 | 0.9974 | 1 / x | 0.02 | 10 |
| $GA_{44}$ | gibberellin | 6.49 | y = 2.16439 x + 0.00350 | 0.99653 | 1 / x | 0.02 | 10 |
| $GA_7$ | gibberellin | 7.64 | y = 1.00553 x + 3.11259e-4 | 0.99373 | 1 / x^2 | 0.01 | 10 |
| $GA_4$ | gibberellin | 7.84 | y = 1.12715 x + 0.03455 | 0.99212 | 1 / x^2 | 0.005 | 10 |
| $GA_{53}$ | gibberellin | 7.95 | y = 1.05812 x + 0.02777 | 0.994 | 1 / x^2 | 0.1 | 100 |
| $GA_9$ | gibberellin | 9.78 | y = 1.38483 x + 7.37642e-4 | 0.99209 | 1 / x^2 | 0.01 | 10 |
| $GA_{15}$ | gibberellin | 9.93 | y = 0.93926 x + 0.04470 | 0.99169 | 1 / x^2 | 0.005 | 10 |
