# Supplementary material for: Gibberellin Homeostasis Perturbation Alters Sugar Metabolism and Developmental Traits in Agapanthus praecox subsp. orientalis
Source: Plants (Basel). 2026 Sep 2;15(17):2699. doi: 10.3390/plants15172699 (PMC13567497; doi:10.3390/plants15172699)
Supplement: Supplementary file 1 [file plants-15-02699-s001.zip › Supplementary Table S11.pdf]

**Table S11: Primer sequences used for qRT-PCR**

| Gene | ID | Forward primer (5'–3') | Reverse primer (5'–3') | Length of products |
|---|---|---|---|---|
| *EXP* | Cluster-49549.0 | CTACCAAGCATTTCAAAC | CTTCCCATCCATAGTTCT | 171 |
| *AGL9* | TRINITY_DN25631_c0_g1 | AATTAAGGATGCTCAGTTAC | CTTCACCAAGAACATTCC | 102 |
| *CDPK* | Cluster-61025.0 | GACAACAGCAATATCAAT | GTATTCTATCAAGTAACTCTC | 164 |
| *MET1* | Cluster-23652.0 | ATTCCCAAGTTAGTATCC | TATAATAGCAAGGCACAT | 156 |
| *XET2* | Cluster-8708.49 | GTGCTGTTTACAATACTA | ATCAAGTGATAGACTGAG | 138 |
| *CYCD3* | Cluster-38281.0 | AATCCATTCCAATTATGTT | ACTTGATGAACCAGATAT | 179 |
| *CDK* | Cluster-42147.10 | TTGAGTATATGGAATGTAAT | AACCTTTATGATGTCTTT | 185 |
| *MET2* | Cluster-53825.1 | TAATGAGATTGATGATGA | GAAGAAGTATAAGGAAGA | 182 |
| *UXS* | Cluster-59655.4 | CGAAGAACTAGCACCGATT | TAAGCGATTCCAAGGCATAA | 133 |
| *CYCLIN/SDS* | Cluster-39086.3 | CGAATGGTTGGTGAAGTATG | TGGTCCTCTATGCTGCTA | 101 |
| *HYG* | TRINITY_DN1152_c0_g1 | TCCACTATCGGCGAGTACTTCT | AGAGCCTGACCTATTGCATCTC | 225 |
| *WOX9* | PB.3484 | CGCCAACGTCTTCTACTGGT | CGGATCCGGCACTAGACTTC | 145 |
| *SNRK* | Cluster-39336.0 | CTGAATACATACGAGTCT | ATTTCTCACCATCAACTA | 150 |
| *AUX/IAA* | Cluster-26820.1 | GTTCACTGCTAAGAGATTG | CTTCCTGTTGATTGTTCC | 76 |
| *IAA13* | Cluster-44652.0 | AGGCTTAACTCCAAGATTA | GAACCAAACACATAGAAGA | 128 |
| *PIN3A* | Cluster-28743.0 | GGTCCTGATGGTTTAACA | GGATCAAACGAGTCATGA | 118 |
| *SAUR76* | PB.22562.1 | CTGGATGAGATTGTGGAG | TACCGAGGGAAATAACTG | 88 |
| *IAA20* | Cluster-29172.1 | CACGATCATGTTGTTACA | TCTCGTTCGTTAATTACTTC | 132 |
| *ARF7* | Cluster-3391.3 | AGGTGTACTGTTCATTCA | GAGGAGACTGAGACATATC | 127 |
| *ERF2* | Cluster-42591.0 | GCATCTCTTAGCCTATATATG | GGACCTGGTAGTGATATC | 142 |
| *GIDIa* | Cluster-54574.0 | CGATGGATAACGAAGCATTA | TGGAATCAGCATTGTATAATCA | 75 |
| *BRH1* | Cluster-36002.1 | GCATCTGCAACTCTATTG | CGATGTGTGAAAGGATGA | 99 |
| *GID1* | Cluster-54574.1 | TGGCAACATTCTCCTTAA | CCTGCATAGTCACAAAATAC | 92 |

| | | | | |
|---|---|---|---|---|
| *AUX22B* | Cluster-13643.3 | CCTCCCATATCTCTTACAA | CTGGTGATGTTCTAAGGA | 73 |
| *BRI1* | Cluster-12322.0 | CTCAGCTTCAATTTCATCA | TCCAGAGACTTGATCTTC | 134 |
| *ETR2* | Cluster-56805.0 | GCCTAACATATCATATCAATTATG | CCCTATAACAAAGAACCATC | 110 |
| *PYL4* | Cluster-17769.0 | GCCTTACATCTCCTCCTC | TGGTCATCTTGGCTGAAG | 74 |
| *GAI* | Cluster-54679.1 | ACTCGTAGAAATGGTGGTACAG | CCTCCGCCTCATCAATCG | 80 |
| *GH3* | PB.5767.1 | ATCGCTCCGCTATCCTCTCT | AAGTCGACGCCCTTTCCTTT | 190 |
| *PIN1* | PB.7573.2 | GCCTTCAACGTGTTGTCCAC | GACTAGAGTGTTCGGGAGCG | 84 |
| *YUCCA4* | PB.9854.1 | AAGCCAATCCTACGTGGCTC | GATGCTAAGGGTGGAGAGGC | 92 |
| *UGPase* | Cluster-15039.3 | GCCCACTTCCTCAGCAGATT | TGCATCCTGTCAGCGAAACT | 226 |
| *SUS* | Cluster-56865.0 | TGGCTCTCCATAGCTCCAGT | TCCAATCTTGGGCGAGTCAC | 236 |
| *INV* | Cluster-33550.0 | TAAGCACCCTTGGAACCGTC | TTACACGCTGGGACGATACG | 236 |
| *AGPase* | Cluster-54452.0 | TTCCACATGCCAAGAGCCAT | GGGTTTTGGAGGAGGTTGCT | 138 |
| *ISA* | Cluster-35036.1 | GGTGGTTTTGCGGACTGTTC | CAAATTGGGCGAGCTTGGTT | 237 |
| *GWD* | Cluster-63444.2 | CTGCAAGGGAGGACTGATGG | GCCTCCTTTCTGGTCACTCC | 226 |
| *SS* | Cluster-51499.2 | CAGCGGGTGCGGATATTTTG | CAAAAGCCCATCCGAAACCG | 171 |
| *SBE* | Cluster-52740.6 | CGGCCAGAGGCTACAACTAC | AGTAGGTAGGTCGGTTGGCT | 160 |
| *α-amylase* | Cluster-23702.2 | AACCGGCTACATGGGAATCG | TCGTCATCTCTCCCACCACT | 210 |
| *PGI* | Cluster-60133.0 | AGTGTCTTGTCGAGCACGTT | CTGCTCTGATTTGCGACACG | 173 |
| *PGM* | Cluster-35890.1 | CTCCTCCGTTGTTGGCTTCC | AAGAGAGAGGGAAGAACGCC | 165 |
| *HXK* | Cluster-56474.1 | AAAAGCGCGTAGGCTTGTTG | CGCTGCCATCCCTTCCTATT | 113 |
| *β-amylase* | Cluster-48978.2 | CTTGGTGCTTCTGGGGAGTT | GACCCCTAGCCCAAAACGAA | 157 |
| *FRK* | Cluster-34539.1 | TTAGTTGGCTTGGGGACAGC | AGGGTTAGCAGAAGACGTGC | 248 |
| *SPP* | Cluster-22966.11 | TCCATGCCGCCGCTATAAAT | TGGTGAAGCTATGGTGCCTG | 246 |
| *SPS* | Cluster-51160.0 | TGGAGGGCCTGTTGACATTC | TGAGGCCATTCTTTCGGCAT | 140 |
| *SODC* | Cluster-43422.0 | CTGTTACCGAGTTCAAAG | CTCTAGTCAAGTCACCAA | 150 |
| *DREB* | Cluster-51152.0 | GAAGAGATGTTTAATGTC | AGAGATGAAGAATGATAG | 155 |

| | | | | |
|---|---|---|---|---|
| *APX* | Cluster-56018.1 | CTTTGACAACACTTATTTC | CATAGTCAGCAAAGAAAG | 152 |
| *CAT* | Cluster-54700.4 | CCATCTAGCGCCTACAGCTC | CCCTGTCAAACTGTGCAAGC | 148 |
| *GPX* | Cluster-51787.0 | TAATGAAGAGATTGTAGAAT | CTTATCAACCAAGAACTT | 178 |
| *POD* | PB.19541.1 | TCATCTACTACTCTTCTTG | TAGCCTCTTTGTTTACTG | 154 |
| *GR* | Cluster-42411.1 | TTGTGTTCTTCGTGGATG | TTAGGCTCAGTCTCATAGTT | 111 |
| *β-actin* | PB.15182.1 | CAGTGTCTGGATTGGAGG | TAGAAGCACTTCCTGTG | 159 |
