# Supplementary material for: Gibberellin Homeostasis Perturbation Alters Sugar Metabolism and Developmental Traits in Agapanthus praecox subsp. orientalis
Source: Plants (Basel). 2026 Sep 2;15(17):2699. doi: 10.3390/plants15172699 (PMC13567497; doi:10.3390/plants15172699)
Supplement: Supplementary file 1 [file plants-15-02699-s001.zip › Supplementary Table S2.pdf]

Table S2: The targeted GA metabolomics (absolute, ng·g⁻¹ FW)

Index	Class	Tissue	Formula	CK-1	CK-2	CK-3	RNAi-1	RNAi-2	RNAi-3	RNAi_GA-1	RNAi_GA-2	RNAi_GA-3
GA_{15}	gibberellin A_{15}	Leaf	$C_{20}H_{26}O_4$	0.610808383	0.666942425	0.552093256	0.539433924	0.620112804	0.655553801	0.135636934	0.337316537	0.206219053
GA_{19}	gibberellin A_{19}	Leaf	$C_{20}H_{26}O_6$	3.18107784	2.73641187	2.95979588	0.699659549	0.597884425	0.658736426	1.15672937	1.01836233	1.1166429
GA_{24}	gibberellin A_{24}	Leaf	$C_{20}H_{26}O_5$	0.969479042	0.982006288	0.840374225	0.507741741	0.51568771	0.538448174	0.312714401	0.323043391	0.332279659
GA_{34}	gibberellin A_{34}	Leaf	$C_{19}H_{24}O_6$	0.453381238	0.421866771	0.419601761	0.629200242	0.520215713	0.527123396	0.225264968	0.245028994	0.248492771
GA_4	gibberellin A_4	Leaf	$C_{19}H_{24}O_5$	13.1881637	10.2919434	12.7901141	3.8896253	2.39877301	3.65919052	16.6203074	15.6734653	18.0457516
GA_{51}	gibberellin A_{51}	Leaf	$C_{19}H_{24}O_5$	0.172946906	0.169045392	0.184845707	0.486847301	0.446382347	0.440159921	0.103294296	0.0908624275	0.102132303
GA_9	gibberellin A_9	Leaf	$C_{19}H_{24}O_4$	1.83796208	1.4114384	1.88251351	0.75136382	0.900480902	0.91287463	0.784101942	0.851733653	0.830081204
GA_{20}	gibberellin A_{20}	Leaf	$C_{19}H_{24}O_5$	0.308908184	0.285224995	0.315419252	0.194574134	0.209479517	0.196237512	0.0900392395	0.109934213	0.122681323
GA_6	gibberellin A_6	Leaf	$C_{19}H_{22}O_6$	2.14115768	1.60660248	2.14844907	7.05513699	5.96334851	5.73794669	6.27305825	6.83691262	6.70695187
GA_7	gibberellin A_7	Tissue	$C_{19}H_{22}O_5$	0.709742515	0.505889173	0.661378827	0.321559226	0.204605185	0.263611056	2.8464199	2.78074385	2.98395722
GA_8	gibberellin A_8	Leaf	$C_{19}H_{24}O_7$	0.240606786	0.248577324	0.221378827	0.232757857	0.239445874	0.208146101	0.37691343	0.40685063	0.470413943
GA_{12}-ald	gibberellin A_{12} aldehyde	Leaf	$C_{20}H_{28}O_3$	0.109427745	0.0952077029	0.104414849	N/A	N/A	N/A	0.0677028722	0.0729146171	0.0766169539
GA_{44}	gibberellin A_{44}	Leaf	$C_{20}H_{26}O_5$	0.306491018	0.328461387	0.322869722	0.0439645447	0.0368428656	0.0393387957	N/A	N/A	N/A
GA_3	gibberellin A_3	Leaf	$C_{19}H_{22}O_6$	N/A	N/A	N/A	1.15395447	0.839936671	0.909214215	1.42722896	1.59096581	1.55666469
GA_{29}	gibberellin A_{29}	Leaf	$C_{19}H_{24}O_6$	2.90269461	2.44831991	2.91540925	4.60227639	4.33071443	4.58673248	N/A	N/A	N/A
GA_1	gibberellin A_1	Leaf	$C_{19}H_{24}O_6$	N/A	N/A	N/A	N/A	N/A	N/A	0.528268608	0.432745451	0.50962963
GA_{53}	gibberellin A_{53}	Leaf	$C_{20}H_{28}O_5$	N/A	N/A	N/A	N/A	N/A	N/A	0.201108617	0.197896621	0.176933452
GA_5	gibberellin A_5	Leaf	$C_{19}H_{22}O_5$	N/A	N/A	N/A	N/A	N/A	N/A	N/A	N/A	N/A

N/A: not available
