# Supplementary material for: Gibberellin Homeostasis Perturbation Alters Sugar Metabolism and Developmental Traits in Agapanthus praecox subsp. orientalis
Source: Plants (Basel). 2026 Sep 2;15(17):2699. doi: 10.3390/plants15172699 (PMC13567497; doi:10.3390/plants15172699)
Supplement: Supplementary file 1 [file plants-15-02699-s001.zip › Supplementary Table S3.pdf]

Table S3: Basic information of RNA-Seq

Sample	Group	Raw Reads	Raw Base(G)	Clean Reads	Clean Base(G)	Error Rate(%)	Q20(%)	Q30(%)	GC Content(%)
CK-1	CK	63930180	9.59	62698018	9.4	0.02	99.36	98.66	46.61
CK-2	CK	70356090	10.55	68041776	10.21	0.02	99.48	98.85	46.63
CK-3	CK	61976824	9.3	60947288	9.14	0.02	99.3	98.55	46.11
RNAi-1	RNAi	68260822	10.24	65949178	9.89	0.02	99.49	98.89	46.3
RNAi-2	RNAi	57855618	8.68	56820368	8.52	0.02	99.27	98.51	46.3
RNAi-3	RNAi	68524778	10.28	66547372	9.98	0.02	99.46	98.83	46.39
RNAi_GA-1	RNAi_GA	69906278	10.49	67547924	10.13	0.02	99.45	98.81	46.98
RNAi_GA-2	RNAi_GA	60282896	9.04	58823318	8.82	0.02	99.29	98.53	46.89
RNAi_GA-3	RNAi_GA	62796052	9.42	60922804	9.14	0.02	99.28	98.51	47.83
