# Supplementary material for: Gibberellin Homeostasis Perturbation Alters Sugar Metabolism and Developmental Traits in Agapanthus praecox subsp. orientalis
Source: Plants (Basel). 2026 Sep 2;15(17):2699. doi: 10.3390/plants15172699 (PMC13567497; doi:10.3390/plants15172699)
Supplement: Supplementary file 1 [file plants-15-02699-s001.zip › Supplementary Table S4.pdf]

Table S4: ***De novo*** **assembly information of RNA-Seq**

Type	Number	Mean Length	N50	N90	Total Bases
Transcript	228325	1063	1709	441	242786971
Unigene	127857	1332	1881	619	170300033
