# Supplementary material for: Gibberellin Homeostasis Perturbation Alters Sugar Metabolism and Developmental Traits in Agapanthus praecox subsp. orientalis
Source: Plants (Basel). 2026 Sep 2;15(17):2699. doi: 10.3390/plants15172699 (PMC13567497; doi:10.3390/plants15172699)
Supplement: Supplementary file 1 [file plants-15-02699-s001.zip › Supplementary Table S5.pdf]

Table S5: Soluble sugar targeted metabolomics data (absolute, mg·g^{-1} FW)

Index	Class	FORMULA	Tissue	WT-1	WT-2	WT-3	RNAi-1	RNAi-2	RNAi-3	RNAi_GA-1	RNAi_GA-2	RNAi_GA-3
Tre	Trehalose	$C_{12}H_{22}O_{11}$	Leaf	0.116876977	0.12469549	0.113274911	0.0821528439	0.0870877049	0.0909168845	0.0744561719	0.084804219	0.0875330317
Xylitol	Xylitol	$C_5H_{12}O_5$	Leaf	0.00485241174	0.00461494118	0.00535230534	0.00385586777	0.00393018443	0.00439878725	0.00502788606	0.00533219488	0.00562584213
D-Ara	D-Arabinose	$C_5H_{10}O_5$	Leaf	0.0240276479	0.0224403922	0.0244008142	0.0269333982	0.0337612705	0.0290318871	0.0242902549	0.0278081366	0.0283949723
Fru	D-Fructose	$C_6H_{12}O_6$	Leaf	3.52115365	3.40756863	3.64486514	10.992105	9.42760246	12.1727967	5.815992	6.90284279	5.64152841
Gal	D-Galactose	$C_6H_{12}O_6$	Leaf	0.687142715	0.700519608	0.734493639	1.10551094	1.22367828	1.16112075	0.497463268	0.535335008	0.487296129
Gal-A	D-Galacturonic acid	$C_6H_{10}O_7$	Leaf	0.0159440676	0.0172490588	0.0167575369	0.0232202236	0.034567418	0.0247809723	0.0144426387	0.0121107182	0.0141043741
Glu	Glucose	$C_6H_{12}O_6$	Leaf	110.036599	111.378431	120.56855	95.6169178	99.3747951	100.727444	79.0974513	89.4882973	82.1711413
Glucuronic-A	D-Glucuronic acid	$C_6H_{10}O_7$	Leaf	0.0514776728	0.0501823529	0.052698626	0.0522599903	0.0499657787	0.0565304757	0.0671458271	0.0612307383	0.071812368
Inositol	Inositol	$C_6H_{12}O_6$	Leaf	1.64830632	1.64187255	1.56775776	1.19946719	1.13879918	1.37615473	1.0090095	1.01550779	1.12843841
Lev	Levoglucosan	$C_6H_{10}O_5$	Leaf	0.15808553	0.153825294	0.182803664	0.152043753	0.16763873	0.181220073	0.169665967	0.188245706	0.226789341
Man	D-Mannose	$C_6H_{12}O_6$	Leaf	0.259649925	0.267880392	0.293486005	0.362362664	0.453209016	0.432600105	0.351934033	0.41390658	0.428842634
Rha	L-Rhamnose	$C_6H_{12}O_5$	Leaf	0.0313752362	0.0313154902	0.0329840204	0.0482393777	0.0463092213	0.0514358599	0.05422009	0.0766531391	0.074966918
Sorbitol	D-Sorbitol	$C_6H_{14}O_6$	Leaf	0.0134482347	0.0129932549	0.0134293537	0.0119340982	0.0113810246	0.0136616205	0.0123830485	0.0143618483	0.0150486878
Arabinitol	D-Arabinitol	$C_5H_{12}O_5$	Leaf	0.0236463451	0.0243429412	0.0192747074	0.0203578026	0.0211432377	0.0184146994	0.0206308846	0.0230493219	0.0284442433
Xylose	D-Xylose	$C_5H_{10}O_5$	Leaf	0.113286723	0.112948039	0.116846209	0.0912427807	0.100481967	0.102767381	0.137510845	0.137893119	0.154881448
Raffinose	Raffinose	$C_{18}H_{32}O_{16}$	Leaf	1.53920438	1.58123922	1.49561934	2.77258143	2.68260246	3.14799791	1.93324538	2.53265696	2.53047763
1-5-Anh	1,5-Anhydroglucitol	$C_6H_{12}O_5$	Leaf	0.0279254102	0.0286984314	0.0374343003	0.0349238697	0.0327661885	0.0359075797	0.0345713143	0.0371600201	0.0389785822
Suc	Sucrose	$C_{12}H_{22}O_{11}$	Leaf	60.1525609	58.4517647	62.9773028	61.2167234	55.3682377	65.7616309	45.90005	43.2805625	48.7394671
Cel	Cellobiose	$C_{12}H_{22}O_{11}$	Leaf	0.111848235	0.117404902	0.101127328	0.193065241	0.204180738	0.22918139	0.266042979	0.277100954	0.262232278
Ribose	D-Ribose	$C_5H_{10}O_5$	Leaf	N/A	N/A	N/A	N/A	N/A	0.000929488761	0.00470892554	0.00370567554	0.00407161388

N/A: not available
