# Supplementary material for: Gibberellin Homeostasis Perturbation Alters Sugar Metabolism and Developmental Traits in Agapanthus praecox subsp. orientalis
Source: Plants (Basel). 2026 Sep 2;15(17):2699. doi: 10.3390/plants15172699 (PMC13567497; doi:10.3390/plants15172699)
Supplement: Supplementary file 1 [file plants-15-02699-s001.zip › Supplementary Table S6.pdf]

Table S6: Calibration parameters of plant hormone targeted metabolomics

Index	Compounds	Class	RT	Equation	r	Weighting	LLOQ	ULOQ
ABA-GE	ABA-glucosyl ester	ABA	4.541383333	y = 0.30423 x + 0.00104	0.99472	1 / x^2	5	500
ABA	Abscisic acid	ABA	5.343183333	y = 0.08833 x - 0.02682	0.99779	1 / x	0.1	500
ABA-ald	Abscisic aldehyde	ABA	5.561133333	y = 0.02266 x + 6.29593e-4	0.99452	1 / x^2	5	500
TRP	L-tryptophan	Auxin	3.49	y = 0.00742 x + 0.00593	0.99892	1 / x	2	10000
TRA	Tryptamine	Auxin	3.773433333	y = 0.16116 x + 0.00333	0.99204	1 / x	0.1	500
OxIAA	2-oxindole-3-acetic acid	Auxin	4.322883333	y = 0.00690 x + 5.66909e-4	0.99848	1 / x	1	500
IAA-Glc	1-O-indol-3-ylacetylglucose	Auxin	4.368466667	y = 0.00181 x + 4.02243e-4	0.99175	1 / x^2	5	10000
IAA-Asp	Indole-3-acetyl-L-aspartic acid	Auxin	4.38	y = 0.02729 x - 0.00301	0.9957	1 / x^2	0.1	500
IAM	3-Indole acetamide	Auxin	4.4305	y = 0.33655 x + 0.00540	0.99323	1 / x^2	0.1	500
IAA-Glu	Indole-3-acetyl glutamic acid	Auxin	4.497916667	y = 0.19831 x - 0.02814	0.99033	1 / x^2	0.1	500
IAA-Gly	Indole-3-acetyl glycine	Auxin	4.5102	y = 0.30321 x - 0.01154	0.99979	1 / x	1	500
ILA	Indole-3-lactic acid	Auxin	4.7307	y = 0.01801 x + 0.00229	0.99732	1 / x^2	2	500
IAA-Ala	N-(3-Indolylacetyl)-L-alanine	Auxin	4.799933333	y = 0.21331 x - 0.00570	0.99945	1 / x	0.1	500
ICA	Indole-3-carboxylic acid	Auxin	4.878183333	y = 0.01739 x - 1.10955e-5	0.99603	1 / x^2	0.5	500
ICAld	Indole-3-carboxaldehyde	Auxin	5.005866667	y = 0.04687 x + 0.00407	0.99274	1 / x^2	0.1	500
IAA	Indole-3-acetic acid	Auxin	5.130616667	y = 0.01235 x + 4.97247e-4	0.99957	1 / x	0.2	500
IA	3-Indoleacrylic acid	Auxin	5.354366667	y = 0.00865 x + 8.03150e-4	0.99671	1 / x	0.2	500
IAA-Val	N-(3-Indolylacetyl)-L-valine	Auxin	5.458966667	y = 0.83797 x + 0.00170	0.99019	1 / x^2	0.1	500
IPA	3-Indolepropionic acid	Auxin	5.546233333	y = 0.03322 x + 2.20614e-4	0.99576	1 / x^2	0.1	500
IAA-Glu-diMe	Indole-3-acetyl-L-glutamic acid dimethyl ester	Auxin	5.6245	y = 0.05877 x - 0.00137	0.99161	1 / x^2	0.5	500
IAA-Trp	Indole-3-acetyl-L-tryptophan	Auxin	5.81045	y = 0.35363 x - 0.00441	0.99013	1 / x^2	0.1	500
IAA-Leu	N-(3-Indolylacetyl)-L-leucine	Auxin	5.8222	y = 1.14789 x - 1.36639e-4	0.99981	1 / x	0.1	500
IBA	Indole-3-butyric acid	Auxin	5.853566667	y = 0.01923 x + 0.02818	0.99346	1 / x^2	0.2	500

IAN	3-Indoleacetonitrile	Auxin	5.9	y = 0.02395 x + 0.00491	0.99103	1 / x^2	0.5	500
IAA-Phe	N-(3-Indolylacetyl)-L-phenylalanine	Auxin	5.918066667	y = 0.63937 x - 0.00306	0.99043	1 / x^2	0.5	500
IAA-Val-Me	Indole-3-acetyl-L-valine methyl ester	Auxin	6.16405	y = 1.62833 x + 0.03611	0.99378	1 / x^2	0.1	500
MEIAA	Methyl indole-3-acetate	Auxin	6.16405	y = 0.05693 x + 2.45871e-5	0.99621	1 / x^2	0.2	500
IAA-Leu-Me	Indole-3-acetyl-L-leucine methyl ester	Auxin	6.498766667	y = 1.90472 x - 0.00100	0.99593	1 / x^2	0.1	500
IAA-Phe-Me	Indole-3-acetyl-L-phenylalanne methyle ester	Auxin	6.548366667	y = 1.26151 x - 9.53124e-4	0.99713	1 / x^2	0.1	500
Indole	Indole	Auxin	N/A	y = 6.69804e-5 x - 1.66035e-4	0.99586	1 / x^2	0.5	500
tZRMP	9-Ribosyl-trans-zeatin 5'-monophosphate	CK	2.8246	y = 0.01126 x - 0.00234	0.99063	1 / x^2	2	500
tZOG	trans-Zeatin-O-glucoside	CK	3.1606	y = 0.11473 x - 6.30642e-4	0.99977	1 / x	0.2	500
cZRMP	cis-Zeatin riboside monophosphate	CK	3.1732	y = 0.02118 x - 0.00539	0.99017	1 / x^2	2	500
tZ	trans-Zeatin	CK	3.25	y = 0.10038 x + 7.54816e-4	0.99808	1 / x^2	0.1	500
tZ9G	trans-Zeatin-9-glucoside	CK	3.27865	y = 0.06098 x + 9.45383e-4	0.99298	1 / x^2	1	500
DZ	Dihydrozeatin	CK	3.307166667	y = 0.05238 x - 0.00144	0.99976	1 / x	0.1	500
DHZ7G	Dihydrozeatin-7-glucoside	CK	3.31	y = 0.34889 x - 4.15714e-5	0.99611	1 / x^2	0.1	500
cZ	cis-Zeatin	CK	3.381016667	y = 0.06060 x + 4.04166e-4	0.99443	1 / x^2	0.1	500
cZ9G	cis-Zeatin-9-glucoside	CK	3.395866667	y = 0.29364 x + 3.93414e-4	0.99002	1 / x^2	0.1	500
DHZROG	Dihydrozeatin-O-glucoside riboside	CK	3.565183333	y = 0.27287 x - 1.51711e-4	0.99153	1 / x	0.1	500
cZROG	cis-Zeatin-O-glucoside riboside	CK	3.59	y = 0.06344 x - 0.01316	0.99536	1 / x^2	0.1	500
pT9G	4-[[(9-beta-D-Glucopyranosyl-9H-purin-6-yl)amino]methyl]phenol	CK	3.662966667	y = 0.09893 x + 5.16300e-4	0.99468	1 / x	0.1	500
tZR	trans-Zeatin riboside	CK	3.662966667	y = 0.16227 x + 5.24805e-4	0.99388	1 / x^2	0.1	500
DHZR	Dihydrozeatin ribonucleoside	CK	3.679133333	y = 0.06799 x - 5.88581e-4	0.99014	1 / x^2	0.1	500
pT	para-Topolin	CK	3.679133333	y = 0.04482 x + 1.21354e-4	0.9988	1 / x^2	0.2	500
cZR	cis-Zeatin riboside	CK	3.75765	y = 0.11438 x - 3.82385e-5	0.9905	1 / x^2	0.1	500
iP7G	N6-Isopentenyl-adenine-7-glucoside	CK	3.789083333	y = 0.23277 x + 5.32987e-4	0.99169	1 / x^2	0.1	500
mT9G	meta-Topolin-9-glucoside	CK	3.8203	y = 0.11158 x + 2.05751e-4	0.99495	1 / x^2	0.1	500
mT	meta-Topolin	CK	3.836016667	y = 0.07614 x + 2.02044e-4	0.99253	1 / x^2	2	500

iPRMP	N-6-iso-pentenyladenosine-5'-monophosphate	CK	3.867716667	y = 0.04910 x - 0.01344	0.99023	1 / x^2	1	500
K9G	Kinetin-9-glucoside	CK	3.883633333	y = 0.16924 x - 4.54077e-5	0.99029	1 / x^2	0.1	500
K	Kinetin	CK	3.899533333	y = 0.05867 x + 2.74727e-4	0.99805	1 / x^2	0.1	500
BAP7G	N6-Benzyladenine-7-glucoside	CK	3.899533333	y = 0.34020 x - 0.00136	0.99041	1 / x^2	0.1	500
pTR	para-Topolin riboside	CK	4.03295	y = 0.13708 x - 0.00184	0.99052	1 / x^2	0.1	500
iP9G	N6-Isopentenyl-adenine-9-glucoside	CK	4.148183333	y = 0.17536 x - 1.10401e-4	0.99031	1 / x^2	0.1	500
oT9G	ortho-Topolin-9-glucoside	CK	4.179583333	y = 0.10536 x - 3.98185e-4	0.99027	1 / x^2	0.1	500
mTR	meta-Topolin riboside	CK	4.179583333	y = 0.16877 x - 4.09430e-4	0.99123	1 / x^2	0.1	500
IP	N6-isopentenyladenine	CK	4.2108	y = 0.04744 x + 2.50561e-4	0.99066	1 / x^2	0.1	500
oT	ortho-Topolin	CK	4.243183333	y = 0.11195 x + 4.36948e-4	0.99346	1 / x^2	0.1	500
KR	Kinetin riboside	CK	4.307633333	y = 0.19817 x + 4.56223e-4	0.99514	1 / x^2	0.1	500
BAP9G	N6-Benzyladenine-9-glucoside	CK	4.33815	y = 0.29105 x + 2.37895e-5	0.99106	1 / x^2	0.1	500
BAP	6-Benzyladenine	CK	4.399	y = 0.12771 x + 0.00248	0.99337	1 / x^2	0.1	500
2CltZ	2-Chloro-trans-zeatin	CK	4.5102	y = 0.08156 x - 0.00338	0.99947	1 / x	0.1	500
oTR	ortho-Topolin riboside	CK	4.5568	y = 0.12997 x + 1.64099e-4	0.99057	1 / x^2	0.1	500
IPR	N6-isopentenyladenosine	CK	4.5719	y = 0.04726 x + 2.48259e-4	0.99026	1 / x^2	0.1	500
2MeScZ	2-Methylthio-cis-zeatin	CK	4.586983333	y = 0.03283 x - 9.08766e-5	0.99353	1 / x^2	0.1	500
2MeScZR	2-Methylthio-cis-zeatin riboside	CK	4.65005	y = 0.10747 x + 4.91012e-4	0.99356	1 / x^2	0.1	500
BAPR	6-Benzyladenosine	CK	4.726716667	y = 0.39838 x + 1.99882e-4	0.99348	1 / x^2	0.1	500
2MeSiPR	2-Methylthio-N6-isopentenyladenosine	CK	5.8067	y = 0.02557 x + 8.91048e-5	0.99595	1 / x^2	0.1	500
2MeSiP	2-Methylthio-N6-isopentenyladenine	CK	5.929433333	y = 0.01794 x + 0.00125	0.9915	1 / x^2	0.1	500
ACC	1-Aminocyclopropanecarboxylic acid	ETH	0.737966667	y = 12361.51074 x + 5.53775e4	0.99656	1 / x	1	500
GA8	Gibberellin A8	GA	3.903	y = 0.10071 x - 0.00125	0.99442	1 / x^2	2	500
GA29	Gibberellin A29	GA	4.054766667	y = 0.00331 x + 0.00293	0.99106	1 / x^2	2	500
GA3	Gibberellin A3	GA	4.51365	y = 0.05944 x + 0.00451	0.99019	1 / x^2	1	500

GA1	Gibberellin A1	GA	4.544833333	y = 0.02603 x + 0.00481	0.99325	1 / x^2	2	500
GA6	Gibberellin A6	GA	4.9529	y = 0.09393 x + 0.01243	0.99795	1 / x^2	1	500
GA19	Gibberellin A19	GA	5.190233333	y = 0.02738 x + 0.00347	0.99715	1 / x^2	5	500
GA5	Gibberellin A5	GA	5.328216667	y = 0.03157 x + 7.37164e-4	0.99644	1 / x^2	5	500
GA20	Gibberellin A20	GA	5.388666667	y = 0.01552 x + 0.00107	0.9959	1 / x	2	500
GA44	Gibberellin A44	GA	5.4336	y = 0.02601 x + 0.00358	0.9901	1 / x^2	5	500
GA34	Gibberellin A34	GA	5.61	y = 0.06436 x + 0.00189	0.99696	1 / x^2	2	500
GA51	Gibberellin A51	GA	5.7331	y = 0.01495 x + 0.00242	0.99412	1 / x^2	5	500
GA53	Gibberellin A53	GA	5.764483333	y = 0.03493 x + 0.00400	0.99044	1 / x^2	2	500
GA7	Gibberellin A7	GA	6.151866667	y = 0.23960 x + 0.00714	0.9971	1 / x^2	1	500
GA4	Gibberellin A4	GA	6.2134	y = 0.05078 x + 0.00328	0.99104	1 / x^2	2	500
GA24	Gibberellin A24	GA	6.39	y = 0.07666 x + 0.00541	0.997	1 / x^2	2	500
GA15	Gibberellin A15	GA	6.908016667	y = 0.03141 x + 0.00104	0.99103	1 / x^2	2	500
GA9	Gibberellin A9	GA	6.9229	y = 0.06152 x + 0.00337	0.99055	1 / x^2	2	500
GA12-ald	Gibberellin A12 aldehyde	GA	7.769466667	y = 0.03451 x + 0.00216	0.99	1 / x^2	5	500
12-OH-JA	12-Hydroxyjasmonic acid	JA	4.82925	y = 0.08996 x + 0.16317	0.9913	1 / x^2	5	500
JA-ACC	Jasmonate-1-aminocyclopropane-1-carboxylic acid	JA	5.384866667	y = 0.12497 x + 0.00565	0.99112	1 / x^2	0.5	500
JA	Jasmonic acid	JA	5.857766667	y = 0.08431 x + 0.00364	0.99477	1 / x	0.2	500
JA-Val	N-[(-)-Jasmonoyl]-(L)-valine	JA	6.028633333	y = 0.78986 x + 0.00103	0.99633	1 / x^2	0.1	500
H2JA	Dihydrojasmonic acid	JA	6.2134	y = 0.06732 x + 0.00425	0.99009	1 / x^2	1	10000
JA-ILE	Jasmonoyl-L-isoleucine	JA	6.356816667	y = 0.26373 x + 2.60235e-6	0.99775	1 / x^2	0.1	500
JA-Phe	N-[(-)-Jasmonoyl]-(l)-phenalanine	JA	6.421783333	y = 0.42099 x + 1.90932e-4	0.99211	1 / x^2	0.1	500
OPC-4	3-oxo-2-(2-(Z)-Pentenyl) cyclopentane-1-butyric acid	JA	6.45185	y = 0.04692 x + 0.01047	0.99037	1 / x^2	2	500
MEJA	Methyl jasmonate	JA	6.963733333	y = 0.49441 x - 0.00862	0.9997	1 / x	0.2	500
OPC-6	3-oxo-2-(2-(Z)-Pentenyl)cyclopentane-1-hexanoic acid	JA	7.060516667	y = 0.10535 x - 0.00402	0.99904	1 / x	5	500
OPDA	cis(+)-12-Oxophytodienoic acid	JA	7.299666667	y = 1.12201 x + 0.06258	0.99108	1 / x^2	0.1	500

MLT	Melatonine	MLT	4.963633333	y = 1.04591e6 x + 3887.49031	0.99004	1 / x^2	0.5	500
Phe	L-Phenylalanine	SA	2.187583333	y = 882.56686 x + 8746.16468	0.9905	1 / x^2	1	15000
SAG	Salicylic acid 2-O-β-glucoside	SA	3.665466667	y = 0.03873 x + 1.61792e-6	0.99036	1 / x^2	1	10000
2-Coumarate	2-Coumarate	SA	4.980966667	y = 3.98235e4 x - 7023.81180	0.99196	1 / x^2	10	500
SA	Salicylic acid	SA	5.17615	y = 0.05320 x + 0.03385	0.99187	1 / x^2	0.5	500
t-CA	trans-Cinnamic acid	SA	5.639183333	y = 255.93132 x - 2810.17083	0.99972	1 / x	200	10000
MeSAG	2-Methoxycarbonylphenyl beta-D-glucopyranoside	SA	N/A	y = 29871.85070 x + 8.17484e4	0.99038	1 / x^2	20	500
ST	(±) Strigol	SL	6.43685	y = 752.37990 x + 717.07405	0.99747	1 / x	20	10000
5DS	5-Deoxystrigol	SL	7.537816667	y = 5915.26579 x + 1993.91399	0.99133	1 / x	2	10000
