# Supplementary material for: Gibberellin Homeostasis Perturbation Alters Sugar Metabolism and Developmental Traits in Agapanthus praecox subsp. orientalis
Source: Plants (Basel). 2026 Sep 2;15(17):2699. doi: 10.3390/plants15172699 (PMC13567497; doi:10.3390/plants15172699)
Supplement: Supplementary file 1 [file plants-15-02699-s001.zip › Supplementary Table S7.pdf]

Table S7: Calibration parameters of soluble sugar targeted metabolomics

Index	Class	RT	Equation	R2	Weighting	LLOQ	ULOQ
2-Deo-ribose	monosaccharide	2.866416667	y = 0.167321 x - 1.061467E-004	0.994438443	1/x	0.029	5
Xylose	monosaccharide	3.571533333	y = 0.416024 x - 8.854073E-005	0.997200317	1/x	0.009	5
D-Ara	monosaccharide	3.637866667	y = 0.477392 x + 4.420211E-005	0.998558439	1/x	0.014	5
Ribose	monosaccharide	3.79095	y = 0.811266 x + 0.002371	0.995365147	1/x	0.021	5
Xylulose	monosaccharide	3.780733333	y = 0.873073 x + 0.005055	0.997827025	1/x	0.021	5
Ribono-1-4-lactone	monosaccharide	3.877683333	y = 0.337751 x + 2.253407E-004	0.999562467	1/x	0.1	5
Xylitol	monosaccharide	4.079083333	y = 1.061554 x - 3.079298E-004	0.998550027	1/x	0.01	5
Lev	monosaccharide	4.129483333	y = 0.214518 x - 9.842067E-005	0.997576629	1/x	0.021	5
Rha	monosaccharide	4.211966667	y = 0.738252 x - 2.624378E-004	0.997175783	1/x	0.048	5
Arabinitol	monosaccharide	4.230283333	y = 1.050558 x - 3.722802E-004	0.99708773	1/x	0.04	5
Fuc	monosaccharide	4.4548	y = 0.087812 x - 1.856200E-005	0.998905895	1/x	0.025	5
Deo	monosaccharide	5.107266667	y = 0.255242 x - 1.130772E-004	0.994624171	1/x	0.032	5
1-5-Anh	monosaccharide	5.88305	y = 0.748664 x - 2.958617E-004	0.994892906	1/x	0.027	5
Met	monosaccharide	5.878583333	y = 0.740989 x - 2.726982E-004	0.994943029	1/x	0.022	5
Fru	monosaccharide	6.0926	y = 0.640867 x - 0.011178	0.996296808	1/x	0.006	50
Man	monosaccharide	6.266483333	y = 0.684305 x - 4.795236E-004	0.993924445	1/x	0.008	5
Gal	monosaccharide	6.324433333	y = 0.547139 x - 8.199301E-004	0.990049192	1/x	0.011	5
Glu	monosaccharide	6.413616667	y = 0.720989 x - 0.001021	0.990079479	1/x	0.011	50
Sorbitol	monosaccharide	6.917416667	y = 2.442241 x - 2.744247E-004	0.991496838	1/x	0.007	50
Glucuronic-A	monosaccharide	6.926333333	y = 0.521338 x - 4.570817E-004	0.996233014	1/x	0.009	5
Gal-A	monosaccharide	7.0378	y = 0.336856 x - 2.107370E-004	0.993751756	1/x	0.012	5
2-Ace-2-Deo-D-Glucosamine	monosaccharide	8.8662	y = 0.038843 x - 2.156546E-005	0.992269188	1/x	0.028	5
Inositol	monosaccharide	8.942083333	y = 0.649270 x - 5.219660E-004	0.994612736	1/x	0.013	25

Ribose-5-pho-Ba	monosaccharide	9.256466667	y = 0.007554 x - 6.095595E-006	0.99370837	1/x	0.074	5
Man-6-pho	monosaccharide	11.35393333	y = 0.001022 x - 5.524831E-007	0.991678708	1/x	0.023	5
Phe	disaccharide	11.43336667	y = 0.454339 x - 2.113073E-004	0.990612105	1/x	0.004	5
Suc	disaccharide	14.18531667	y = 2.191238 x - 0.175824	0.993392191	1/x	0.007	50
Lac	disaccharide	14.56425	y = 0.042421 x - 2.292887E-005	0.994727753	1/x	0.019	5
Cel	disaccharide	14.70763333	y = 0.203059 x - 1.623232E-004	0.990453776	1/x	0.015	25
Mal	disaccharide	14.93295	y = 0.187744 x - 1.428511E-004	0.992376009	1/x	0.029	25
Tre	disaccharide	14.9944	y = 1.231090 x - 8.720645E-004	0.990790667	1/x	0.003	25
Raffinose	trisaccharide	20.73661667	y = 0.200314 x - 3.556551E-005	0.998598723	1/x	0.021	25
