# Supplementary material for: Gibberellin Homeostasis Perturbation Alters Sugar Metabolism and Developmental Traits in Agapanthus praecox subsp. orientalis
Source: Plants (Basel). 2026 Sep 2;15(17):2699. doi: 10.3390/plants15172699 (PMC13567497; doi:10.3390/plants15172699)
Supplement: Supplementary file 1 [file plants-15-02699-s001.zip › Supplementary Table S8.pdf]

Table S8: Information of reference external standard

Index	Compounds	Class	Q1 (Da)	Q3 (Da)	Molecular Weight	Ion mode	Ionization model	Formula	Brand	Catalog No.	Corresponding internal standard
GA_{15}	gibberellin A_{15}	gibberellin	430.1	371.1	330.18311	[M]+	positive	C20H26O4	Analytika	0122442	IS-GA15
GA_{19}	gibberellin A_{19}	gibberellin	281.3	118.2	362.17294	[M]2+	positive	C20H26O6	Analytika	0122692	IS-GA1
GA_{24}	gibberellin A_{24}	gibberellin	273.2	243.7	346.178025	[M]2+	positive	C20H26O5	Analytika	0122422	IS-GA1
GA_{34}	gibberellin A_{34}	gibberellin	448.1	389.1	348.15729	[M]+	positive	C19H24O6	Analytika	0122401	IS-GA34
GA_{4}	gibberellin A_{4}	gibberellin	432.4	373.3	332.162375	[M]+	positive	C19H24O5	Analytika	0122531	IS-GA4
GA_{51}	gibberellin A_{51}	gibberellin	432.1	373.1	332.162375	[M]+	positive	C19H24O5	Analytika	0122352	IS-GA20
GA_{9}	gibberellin A_{9}	gibberellin	416	357.3	316.16746	[M]+	positive	C19H24O4	Analytika	0122662	IS-GA9
GA_{20}	gibberellin A_{20}	gibberellin	432.4	373.2	332.162375	[M]+	positive	C19H24O5	Analytika	0122482	IS-GA20
GA_{6}	gibberellin A_{6}	gibberellin	446	387	346.14164	[M]+	positive	C19H22O6	TRC	G377320	IS-GA6
GA_{7}	gibberellin A_{7}	gibberellin	430.3	371.3	330.146725	[M]+	positive	C19H22O5	Analytika	0122542	IS-GA4
GA_{8}	gibberellin A_{8}	gibberellin	464.3	405.2	364.152205	[M]+	positive	C19H24O7	zzstandard	ZC-25306	IS-GA8
GA_{12}-ald	gibberellin A_{12} aldehyde	gibberellin	416.2	253.2	316.203845	[M]+	positive	C20H28O3	Analytika	0122682	IS-GA1
GA_{44}	gibberellin A_{44}	gibberellin	446.3	387.2	346.178025	[M]+	positive	C20H26O5	Analytika	0122360	IS-GA1
GA_{3}	gibberellin A_{3}	gibberellin	446.3	387.2	346.14164	[M]+	positive	C19H22O6	Analytika	0122501	IS-GA1
GA_{29}	gibberellin A_{29}	gibberellin	448	389	348.15729	[M]+	positive	C19H24O6	zzstandard	ZC-25804	IS-GA29
GA_{1}	gibberellin A_{1}	gibberellin	448.3	389.2	348.15729	[M]+	positive	C19H24O6	Analytika	0122492	IS-GA1
GA_{53}	gibberellin A_{53}	gibberellin	448.3	389.2	348.193675	[M]+	positive	C20H28O5	Analytika	012 2343	IS-GA53
