# Supplementary material for: Gibberellin Homeostasis Perturbation Alters Sugar Metabolism and Developmental Traits in Agapanthus praecox subsp. orientalis
Source: Plants (Basel). 2026 Sep 2;15(17):2699. doi: 10.3390/plants15172699 (PMC13567497; doi:10.3390/plants15172699)
Supplement: Supplementary file 1 [file plants-15-02699-s001.zip › Supplementary Table S9.pdf]

Table S9: Information of reference internal standard

IS name	Compounds	Brand	Catalog No.
IS-GA_1	[2H2]GIBBERELLIN A1	Analytika	0322493
IS-GA_{15}	[2H2]GIBBERELLIN A15	isoreag	IR-35471
IS-GA_{20}	[2H2]GIBBERELLIN A20	Analytika	0322483
IS-GA_{29}	[2H2]GIBBERELLIN A29	Analytika	0322471
IS-GA_{34}	[2H2]GIBBERELLIN A34	Analytika	0322403
IS-GA_4	[2H2]GIBBERELLIN A4	Analytika	0322533
IS-GA_{53}	[2H2]GIBBERELLIN A53	Analytika	0322343
IS-GA_6	[2H2]GIBBERELLIN A6	Analytika	0322643
IS-GA_8	[2H2]GIBBERELLIN A8	Analytika	0322651
IS-GA_9	[2H2]GIBBERELLIN A9	Analytika	0322661
IS-GA_1	[2H2]GIBBERELLIN A1	Analytika	0322493
IS-GA_{15}	[2H2]GIBBERELLIN A15	isoreag	IR-35471
